# Integrated Network Analysis of Symptom Clusters Across Monkeypox Epidemics From 1970 to 2023: Systematic Review and Meta-Analysis

**DOI:** 10.2196/49285

**Published:** 2024-02-16

**Authors:** Shu Su, Meng Jia, Yingni Yu, Hu Li, Wenwei Yin, Yi Lu, Rongzhong Huang, Rong Xiang, Huizhe Huang, Peng Hu

**Affiliations:** 1 Key Laboratory of Molecular Biology for Infectious Diseases Department of Infectious Diseases The Second Affiliated Hospital of Chongqing Medical University Chongqing China

**Keywords:** monkeypox, mpox, symptoms, prevalence, meta-analysis, network analysis, evolution trends

## Abstract

**Background:**

The worldwide spread of monkeypox (mpox) has witnessed a significant increase, particularly in nonendemic countries.

**Objective:**

We aimed to investigate the changing clinical symptoms associated with mpox from 1970 to 2023 and explore their interrelations.

**Methods:**

In this systematic review and meta-analysis, 3 electronic databases were searched for English peer-reviewed studies conducted from January 1970 to April 2023 that reported any symptoms among confirmed mpox cases. We categorized the mpox epidemics into 3 periods: 1970-2002 (period 1, within the African region), 2003-2021(period 2, epidemics outside Africa), and 2022-2023 (period 3, worldwide outbreak). Following PRISMA guidelines, a meta-analysis was performed to estimate the pooled prevalence for each symptom. The correlation among symptoms was analyzed and visualized using network analysis.

**Results:**

The meta-analysis included 61 studies that reported 21 symptoms in 720 patients from period 1, 39 symptoms in 1756 patients from period 2, and 37 symptoms in 12,277 patients from period 3. The most common symptom among patients from all 3 periods was rash (period 1: 92.6%, 95% CI 78.2%-100%; period 2: 100%, 95% CI 99.9%-100%; and period 3: 94.8%, 95% CI 90.9%-98.8%), followed by lymphadenopathy (period 1: 59.8%, 95% CI 50.3%-69.2%; period 2: 74.1%, 95% CI 64.2%-84.1%; and period 3: 61.1%, 95% CI 54.2%-68.1%). Fever (99%, 95% CI 97%-100%), enlarged lymph nodes (80.5%, 95% CI 75.4%-85.0%), and headache (69.1%, 95% CI 4%-100%) were the main symptoms in period 1, with a significant decrease in period 3: 37.9%, 31.2%, and 28.7%, respectively. Chills/rigors (73.3%, 95% CI 60.9%-85.7%), fatigue (68.2%, 95% CI 51.6%-84.8%), and dysphagia/swallowing difficulty (61.2%, 95% CI 10.5%-100%) emerged as primary new symptoms in period 2 and decreased significantly in period 3. Most other symptoms remained unchanged or decreased in period 3 compared to the former 2 periods. Nausea/vomiting had the highest degree of correlation (with 13 symptoms) and was highly positively correlated with lymphadenopathy (r=0.908) and conjunctivitis (r=0.900) in period 2. In contrast, rash and headache were 2 symptoms with the highest degree of correlation (with 21 and 21 symptoms, respectively) in period 3 and were highly positively correlated with fever (r=0.918 and 0.789, respectively).

**Conclusions:**

The manifestation of symptoms in patients with mpox has become more diverse, leading to an increase in their correlation. Although the prevalence of rash remains steady, other symptoms have decreased. It is necessary to surveil the evolving nature of mpox and the consequential changes in clinical characteristics. Epidemic countries may shift their focus on the potential association among symptoms and the high synergy risk.

**Trial Registration:**

PROSPERO Registration: CRD42023403282; http://tinyurl.com/yruuas5n

## Introduction

### Background

Monkeypox (mpox), caused by the mpox virus within the genus *Orthopoxvirus* (family Poxviridae, subfamily Chordopoxvirinae), is an emerging zoonotic disease that results in a smallpox-like illness in humans [1,2]. The first case of mpox was reported in Zaire (current Democratic Republic of the Congo, DRC) in 1970, after which it spread to other countries in Central and West Africa [1,3]. Although mpox has predominantly affected African populations, in the spring of 2003, the disease was reported outside Africa for the first time in the Midwest region of the United States due to infection from imported animals, and then sporadic reports of infections emerged from other countries caused by imported cases from West Africa until the pandemic [4]. Since May 2022, the disease has emerged as a major worldwide public health crisis, with 87,042 cases reported worldwide by April 30, 2023, mainly among men who have sex with men (MSM) [5]. The potential worldwide impact of mpox necessitates an urgent need for an improved understanding of the disease’s epidemiology, symptomatology, and management.

Prior to the 2022 worldwide pandemic, mpox was considered a rare disease with a low case fatality rate (CFR, <10%) in comparison to its closely related virus, variola major (CFR=30%) [6]. Despite this, mpox remained a serious health threat in the DRC and other countries in Western and Central Africa [7], particularly among the pediatric population and patients with immunodeficiency [8]. The incubation period of mpox ranges from 5 to 21 days and is characterized by nonspecific symptoms, such as fever, headache, and lymphadenopathy [9]. Skin lesions were observed in 95% of cases, with rashes being the most common symptom, typically appearing on the face and extremities within 1-3 days after the onset of fever [10,11]. The diverse range and frequency of the clinical symptoms of mpox are influenced by various risk factors, including modes of transmission and demographics of affected populations [12]. For example, since 2022, the majority of mpox cases have occurred among MSM, resulting in comorbidity with other infectious diseases, such as HIV and syphilis [13]. An improved understanding of the clinical profile of mpox can aid in the development of effective strategies for managing and responding to outbreaks of the disease.

Before the onset of the pandemic in 2022, limited and outdated data existed regarding the epidemiology and clinical characteristics of mpox from an epidemiological perspective [14-16]. However, since the emergence of the worldwide mpox pandemic in 2022, numerous reviews and meta-analyses have begun to investigate the symptoms of mpox, although most focus only on 2022 [17-20]. Only 2 papers have compared mpox symptom prevalence before and after 2022, with limitations including sparse literature, especially prior to 2010 [21,22], and a lack of correlation analyses between symptoms. Therefore, there is a need for a comprehensive longitudinal analysis to investigate the evolution of mpox symptoms and their changes over time.

### Objective

To address this gap, we conducted a review and meta-analysis to comparatively analyze mpox symptom prevalence and correlations during 3 periods: 1970-2002 in Africa; 2003-2021 in Africa, Europe, and North America; and 2022-2023 worldwide.

## Methods

### Protocol Registration

This research adheres to the methodological recommendations of the Cochrane systematic review methodology guidance [23] and conforms to the guidelines of the PRISMA (Preferred Reporting Items for Systematic Reviews and Meta-Analyses) statement (see the PRISMA checklist in Table S9 in Multimedia Appendix 1) [24]. This study has been registered with PROSPERO (International Prospective Register of Systematic Reviews; registration number CRD42023403282).

### Search Strategy

Two independent investigators (authors SS and MJ) conducted a comprehensive literature search of peer-reviewed research papers published from January 1970 to April 2023 in the following English literature databases: PubMed, Web of Science, and ScienceDirect. We also searched all mpox-related public databases. Keywords pattern in the database search were used through combinations of monkeypox (eg, “monkeypox” OR “monkeypox virus” OR “human monkeypox”), symptoms (eg, “symptoms” OR “characteristics” OR “clinical characteristics” OR “clinical symptoms”), or cases (eg, “cases” OR “cases report”) and excluded reviews. Full details of the search strategy are listed in Table S1 in Multimedia Appendix 1. A manual search of the reference lists of the published papers was also performed.

We restricted our search to research conducted on humans with observational studies, discarding veterinary investigations. Papers were included if they contained clinical or epidemiological information relevant to the current monkeypox virus outbreak or historical information to establish context. The following types of publications were excluded: news reports, conference abstracts, mathematical modeling studies, dissertations, and studies with mpox self-reported prevalence. Two independent investigators (SS and MJ) confirmed the eligibility of each study; if necessary, a third senior reviewer (author PH) resolved differences. The PRISMA flow diagram was applied to report the paper search process.

The following information was collected from each of the studies: authors, publication year, title, study design, study period, study area, sample size, study population, age, and symptoms. The prevalence of each symptom was calculated based on the percentage of patients with mpox who had that symptom. Discrepancies in data extraction between the 2 reviewers (SS and MJ) were adjudicated in the final meeting by the third senior reviewer (PH). Data were extracted and stored in Microsoft Excel for analysis.

### Quality Assessment

Two reviewers (SS and MJ) independently assessed the study quality using the Risk of Bias in Nonrandomized Studies - of Interventions (ROBINS-I) tool for nonrandomized controlled trial assessment. At the protocol stage, we specified the review question of participants as confirmed patients with mpox and outcomes as any symptom report. Additionally, we listed the confounding domains relevant to all studies, including time-varying confounding and baseline confounding, and the cointerventions that could be different between intervention groups and that could impact outcomes, such as any treatment. We assessed the overall risk of bias according to 7 items: bias due to confounding, bias in the selection of participants for the study, bias in the classification of interventions, bias due to deviations from intended interventions, bias due to missing data, bias in the measurement of outcomes, and bias in the selection of the reported result. Each item was classified as low risk, moderate risk, serious risk, or critical risk of bias. The overall risk of bias was assessed according to the results of the 7 items [25]. The detailed results are shown in Table S2 in Multimedia Appendix 1.

### Study Period

In this review, we classified the mpox epidemics into 3 distinct periods. Period 1, spanning from 1970 to 2002, was characterized by the limited transmission of the virus within the African region. Period 2, spanning from 2003 to 2021, marked the first outbreak of mpox outside Africa, with confirmed cases reported in the United States, the United Kingdom, Singapore, and Israel. Period 3, from 2022 to 2023, witnessed an unprecedented worldwide explosion of mpox cases.

### Meta-Analysis

To estimate the prevalence of mpox symptoms, we conducted meta-analyses by combining data from various studies using random effects models that fitted study-specific effects. We calculated corresponding 95% CIs. Heterogeneity analysis was performed to quantify the inconsistency among results, and it was expressed using I^2^, a quantity representing the degree of heterogeneity from 0% to 100%. Therefore, I^2^ with experience values of 25%, 50%, and 75% indicated low, moderate, and high heterogeneity, respectively [26]. The meta-analyses were conducted using R Studio software (version 2023.06.2, build 561), which is a specialized integrated development environment (IDE) designed for R programming (version 4.2.1, R Foundation for Statistical Computing).

### Sensitivity Analysis

To assess the impact of bias on our findings, we conducted a sensitivity analysis by excluding studies with a moderate and higher risk of bias, thereby including only low-risk studies in a subgroup meta-analysis.

### Subgroup Analysis

To assess the impact of geographical differences on our findings, we conducted a subgroup analysis by grouping patients based on the regional distribution of the World Health Organization (WHO). *P*≤.05 was considered a statistically significant difference in prevalence among regions.

### Network Analysis

We also conducted a network analysis to compare changes in mpox symptoms across the 3 periods and created nodes (symptoms) and edges (symptom correlation). Within each period, the edge color reflected the correlation properties, where pink color represented positive correlation; thickness indicated the correlation strength between symptoms, where correlations were calculated through Spearman intragroup correlation analyses using R software v4.2.1 and filtered significantly correlated (*P*≤.05) symptom pairs. Similarly, the size and color depth of the nodes represented degrees, which were acquired through clustering analyses of a subset of nodes and visualized using Cytoscape software (v3.7.2).

## Results

### Overview

**Figure 1:**
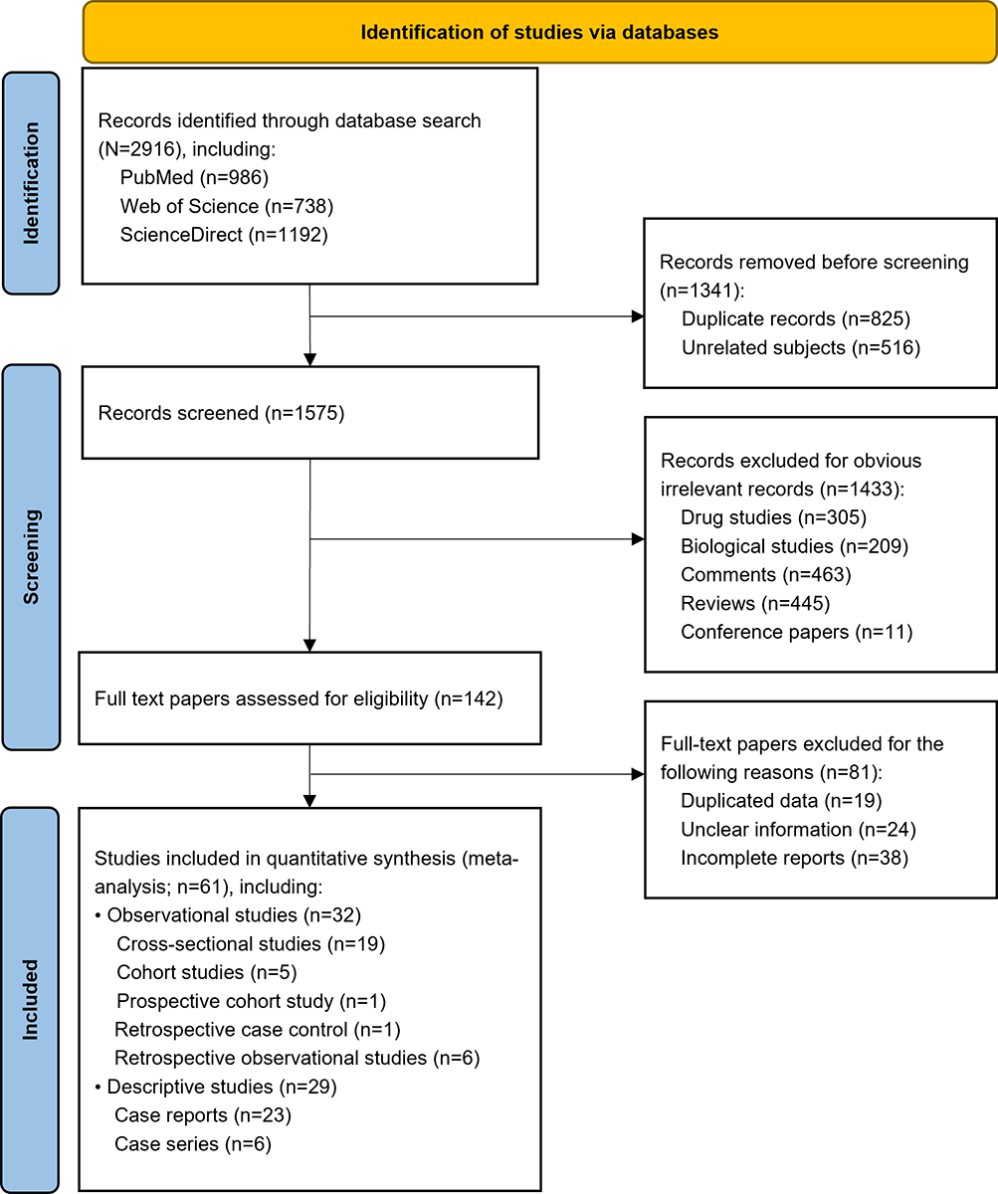
Study selection flowchart.

The initial search strategy included 2916 unique titles. After excluding 825 (28.3%) duplicate records and 516 (17.7%) unrelated subjects, 1575 (54%) studies remained. Of these, 142 (9%) full-text papers were retrieved. The majority were excluded for reasons such as being drug or biological studies, comments, reviews, or conference papers. After reviewing the 142 papers, 19 (13.4%) were excluded due to duplicate data, while 24 (16.9%) and 38 (26.8%) contained unclear or incomplete information, respectively. Additionally, 19 (13.4%) papers presented the same outbreak with duplicate data. Ultimately, our systematic review and meta-analysis included 61 (43%) papers that provided information about cases and symptoms (Figure 1). Of these, 29 (47.5%) papers were assessed as having low risk of bias, 23 (37.7%) were assessed as having moderate risk of bias, 4 (6.6%) exhibited a serious risk of bias, while the remaining 5 (8.2%) were categorized as having a critical risk of bias (Table S2 in Multimedia Appendix 1).

From 1970 to 2002 (period 1), all 720 patients from Africa were included in our meta-analysis (n=4, 6.6%, studies). The median age was 5.4 years (IQR 2.7-9.8; from n=3, 75%, of 4 studies). The proportion of male patients was 34.9% (n=251, range 15.9-56.8%; from n=4, 6.6%, studies).

From 2003 to 2021 (period 2), a total of 12 (19.7%) studies were included. Most of the 1756 patients were also from Africa (n=1259, 71.7%, patients from n=5, 41.7%, studies), followed by the Americas (n=457, 26%, patients from n=3, 25%, studies), Europe (n=5, 0.3%, patients from n=2, 16.7%, studies), and the Western Pacific (n=2, 0.1%, patients from n=2, 16.7%, studies). The median age was 21.7 years (IQR 13.6-32.1; from n=8, 66.7%, studies), and the proportion of male patients was 53.3% (n=934; from n=11, 91.7%, studies).

From 2022 to 2023 (period 3), 45 (73.7%) studies involving 12,277 patients were included in our study. A total of 9587 (78.1%) patients were reported in the Americas (n=13, 28.9%, studies), followed by Europe (n=1680, 13.7%, patients from n=24, 5.3%, studies). The median age was 34.5 years (IQR 28.5-42.0; from n=45, 100%, studies), and the proportion of male patients was 86.7% (n=10,640; from n=45, 100%, studies). Among the male patients, the proportion of MSM was 88.8% (n=2340; from n=27, 60%, studies), with HIV (n=2116, 37.8%; from n=30, 66.7%, studies), syphilis (n=125, 18.4%; from n=9, 20%, studies), and other sexually transmitted infections (STIs; n=221, 23.6%; from n=6, 13.3%, studies). The study details are given in Table 1. The period prevalence was calculated for each period (Figure S1 in Multimedia Appendix 1).

**Table 1:** Characteristics of the included studies (N=61).

Characteristic			Period 1 (1970-2002)				Period 2 (2003-2021)					Period 3 (2022-2023)		
			Studies, n (%)	Patients		Studies, n (%)			Patients		Studies, n (%)			Patients
Overall, n (%)			4 (6.6)	720 (4.9)		12 (19.7)			1756 (11.9)		45 (73.8)			12,277 (83.2)
**Study type, n (%)**														
	Observational	2 (50.0)		370 (51.4)	7 (58.3)			1748 (99.5)		23 (51.1)			11,502 (93.7)	
	Cross-sectional	0		0	4 (33.3)			550 (31.3)		15 (33.3)			10,610 (86.4)	
	Cohort	0		0	0			0		5 (11.1)			704 (5.7)	
	Prospective cohort	0		0	0			0		1 (2.2)			77 (0.6)	
	Retrospective case control	0		0	0			0		1 (2.2)			70 (0.6)	
	Retrospective observational	2 (50.0)		370 (51.4)	3 (25.0)			1198 (68.2)		1 (2.2)			41 (0.3)	
	Descriptive	2 (50.0)		350 (48.6)	5 (41.7)			8 (0.5)		22 (48.9)			775 (6.3)	
	Case report	1 (25.0)		6 (0.8)	5 (41.7)			8 (0.5)		17 (37.8)			32 (0.3)	
	Case series	1 (25.0)		344 (47.8)	0			0		5 (11.1)			743 (6.0)	
**Regions, n (%)**														
	Africa	4 (100.0)		720 (100.0)	5 (41.7)			1259 (71.7)		0			0	
	Americas	0		0	3 (25.0)			457 (26.0)		13 (28.9)			9587 (78.1)	
	Eastern Mediterranean	0		0	0			0		2 (4.4)			9 (0.1)	
	Europe	0		0	2 (16.7)			5 (0.3)		24 (53.3)			1680 (13.7)	
	Western Pacific	0		0	2 (16.7)			2 (0.1)		2 (4.4)			10 (0.1)	
**Patients’ characteristics**														
	Age (years), median (IQR)	3 (75.0)		5.4 (2.7-9.8); 376 (52.2)^a^	8 (66.7)			21.7 (13.6-32.1); 1731 (98.6)^a^		45 (100.0)			34.5 (28.5-42.0); 12,277 (100.0)^a^	
	Male, n (%)	4 (100.0)		251 (34.9)^b^	11 (91.7)			934 (53.2)^b^		45 (100.0)			10,640 (86.7)^b^	
	MSM^c^, n (%)	0		0^d^	0			0^d^		27 (60.0)			2340 (88.8)^d^	
	HIV, n (%)	0		0^e^	2 (16.7)			11 (18.0)^e^		31 (68.9)			2116 (37.8)^e^	
	Syphilis, n (%)	0		0^e^	0			0^e^		9 (20.0)			125 (18.4)^e^	
	Other STIs^f^, n (%)	0		0^e^	0			0^e^		6 (13.3)			221 (23.6)^e^	

^a^Patients in the included studies.

^b^Male patients in the included studies.

^c^MSM: men who have sex with men.

^d^MSM patients in the included studies.

^e^Patients with infection/disease in the included studies.

^f^STI: sexually transmitted infection.

### Clinical Symptom Prevalence

**Figure 2:**
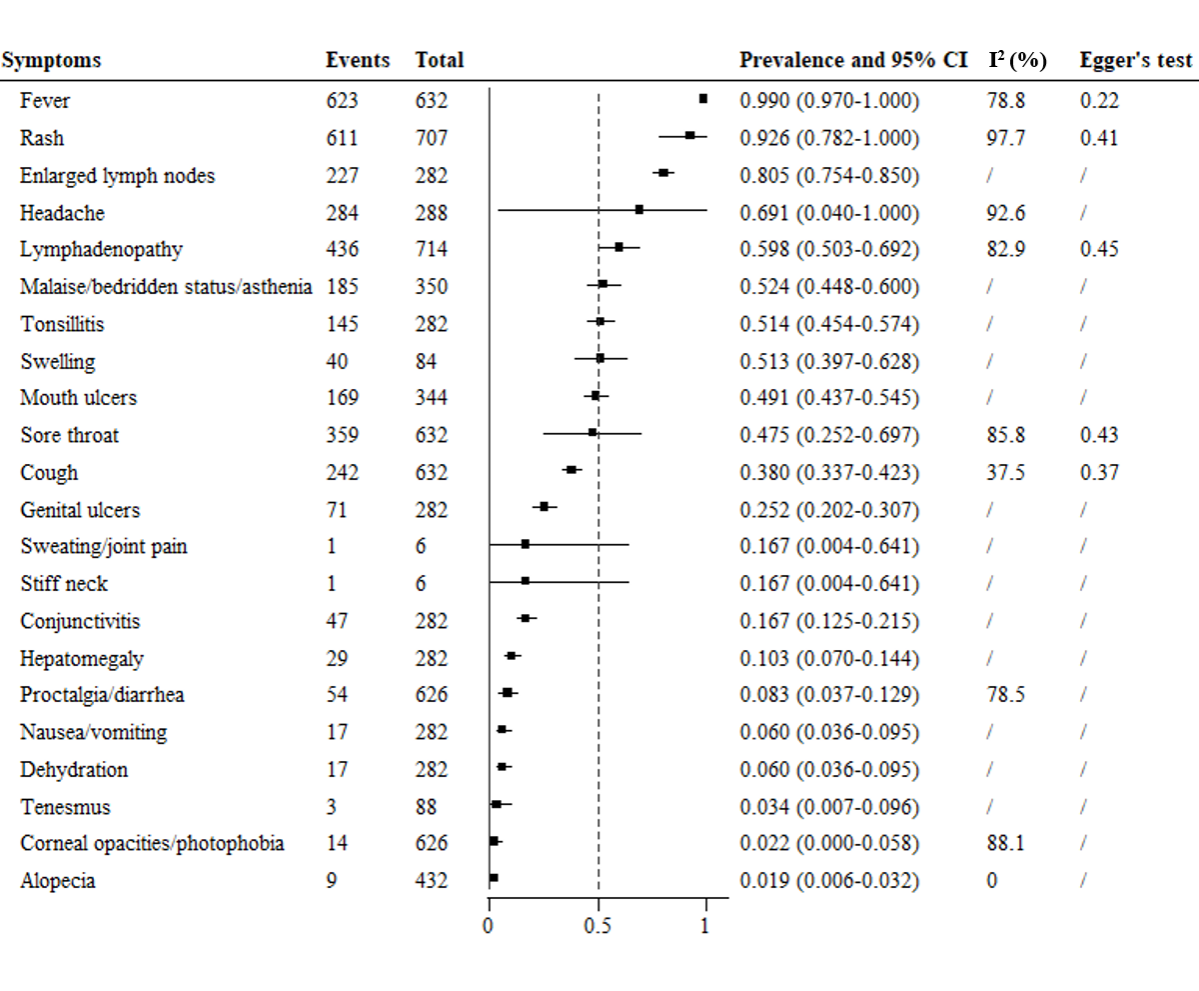
Meta-analysis of the prevalence of clinical symptoms among mpox patients from 1970 to 2002. mpox: monkeypox.

We conducted a meta-analysis of 21 symptoms reported from 1970 to 2002 (Figure 2). The most commonly reported symptoms were fever (99%, 95% CI 97%-100%), rash (92.6%, 95% CI 78.2%-100.0%), and enlarged lymph nodes (80.5%, 95% CI 75.4%-85.0%). These were followed by headache (69.1%, 95% CI 4%-100%), lymphadenopathy (59.8%, 95% CI 50.3%-69.2%), malaise/bedridden status/asthenia (52.4%, 95% CI 44.8%-60.0%), tonsillitis (51.4%, 95% CI 45.4%-57.4%), swelling (51.3%, 95% CI 39.7%-62.8%), mouth ulcers (49.1%, 95% CI 43.7%-54.5%), sore throat (47.5%, 95% CI 25.2%-69.7%), cough (38%, 95% CI 33.7%-42.3%), genital ulcers (25.2%, 95% CI 20.2%-30.7%), sweating/joint pain (16.7%, 95% CI 0.4%-64.1%), stiff neck (16.7%, 95% CI 0.4%-64.1%), conjunctivitis (16.7%, 95% CI 12.5%-21.5%), and hepatomegaly (10.3%, 95% CI 7.0%-14.4%). The prevalence of proctalgia/diarrhea, nausea/vomiting, dehydration, tenesmus, corneal opacity/photophobia, and alopecia was relatively low (<10%).

**Figure 3:**
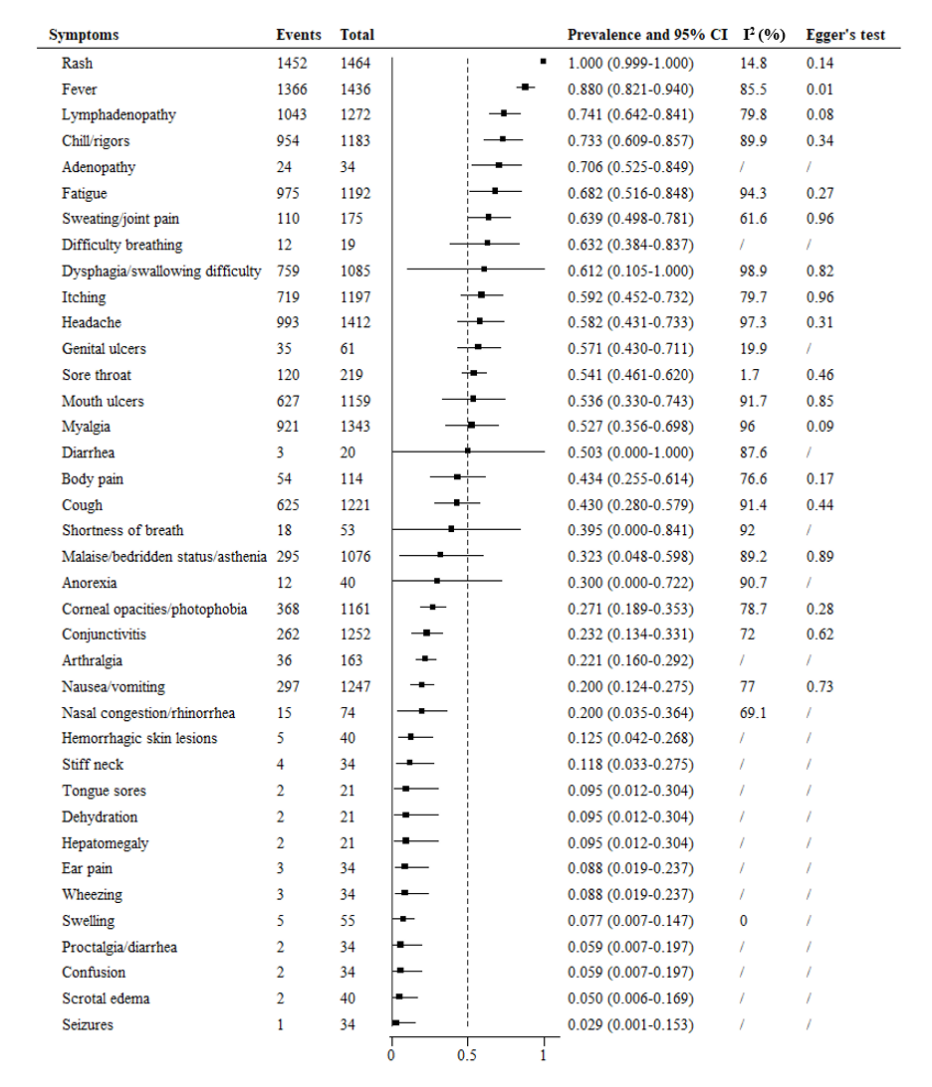
Meta-analysis of the prevalence of clinical symptoms among mpox patients from 2003 to 2021.

In our meta-analysis covering the period of 2003-2021, we identified 38 symptoms associated with mpox (Figure 3). Rash was the most prevalent symptom (100%, 95% CI 99.9%-100%), followed by fever (88%, 95% CI 82.1%-94.0%) and lymphadenopathy (74.1%, 95% CI 64.2%-84.1%). The former decreased, while the latter increased. Chills/rigors (73.3%, 95% CI 60.9%-85.7%), adenopathy (70.6%, 95% CI 52.5%-84.9%), fatigue (68.2%, 95% CI 51.6%-84.8%), difficulty breathing (63.2%, 95% CI 38.4%-83.7%), dysphagia/difficulty swallowing (61.2%, 95% CI 10.5%-100%), itching (59.2%, 95% CI 45.2%-73.2%), myalgia (52.7%, 95% CI 35.6%-69.8%), and diarrhea (50.3%, 95% CI 0%-100%) were newly reported but remained highly prevalent. The prevalence of genital ulcers (57.1%, 95% CI 43.0%-71.1%) increased compared to the period of 1970-2002, while sweating/joint pain (63.9%, 95% CI 49.8%-78.1%), headache (58.2%, 95% CI 43.1%-73.3%), sore throat (54.1%, 95% CI 46.1%-62.0%), mouth ulcers (53.6%, 95% CI 33.0%-74.3%), and cough (43%, 95% CI 28.0%-57.9%) remained steady. The prevalence of body pain (43.4%, 95% CI 25.4%-61.4%), shortness of breath (39.5%, 95% CI 0%-84.1%), anorexia (30%, 95% CI 0.0%-72.2%), corneal opacity/photophobia (27.1%, 95% CI 18.9%-35.3%), arthralgia (22.1%, 95% CI 16.0%-29.2%), nasal congestion/rhinorrhea (20%, 95% CI 3.5%-36.4%), and hemorrhagic skin lesions (12.5%, 95% CI 4.2%-26.8%) was newly reported but was <50%. The prevalence of malaise/bedridden status/asthenia (32.3%, 95% CI 4.8%-59.8%) conjunctivitis (23.2%, 95% CI 13.4%-33.1%), and stiff neck (11.8%, 95% CI 3.3%-27.5%) remained steady compared to the former period, while nausea/vomiting (20%, 95% CI 12.4%-27.5%) increased. The prevalence of 10 (26.3%) other symptoms was <10%, of which 7 (70%) were newly reported symptoms: tongue sores, dehydration, ear pain, wheezing, confusion, scrotal edema, and seizures. Swelling, which was reported from 1970 to 2002, had a decreased prevalence of 7.7% (95% CI 0.7%-14.7%), while hepatomegaly (9.5%, 95% CI 0%-22.7%), swelling, and proctalgia/diarrhea (5.9%, 95% CI 0.7%-19.7%) remained steady in this period.

**Figure 4:**
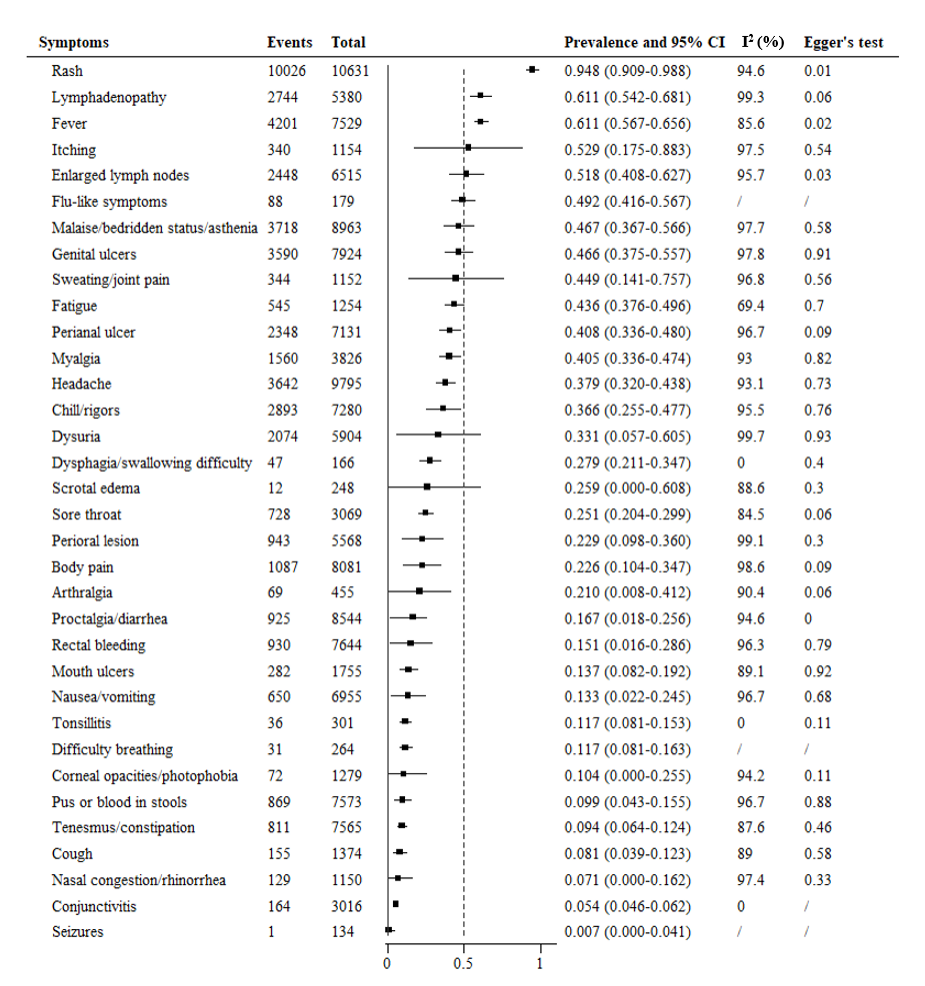
Meta-analysis of the prevalence of clinical symptoms among mpox patients from 2022 to 2023. mpox: monkeypox.

Since 2022, the included literature reports have listed 37 clinic characteristics observed in patients during the mpox pandemic (Figure 4). Rash was the most prevalent symptom among patients, and its prevalence (94.8%, 95% CI 90.9%-98.8%) was higher than that of other symptoms, similar to the 2003-2021 period, followed by fever (61.1%, 95% CI 56.7%-65.6%), enlarged lymph nodes (51.8%, 95% CI 40.8%-62.7%), headache (37.9%, 95% CI 32.0%-43.8%), and chills/rigors (36.6%, 95% CI 25.5%-47.7%), which were lower than the periods from 2003 to 2021. Furthermore, flu-like symptoms (49.2%, 95% CI 41.6%-56.7%), perianal ulcer (40.8%, 95% CI 33.6%-48.0%), dysuria (33.1%, 95% CI 5.7%-60.5%), perioral lesion (22.9%, 95% CI 9.8%-36.0%), rectal bleeding (15.1%, 95% CI 1.6%-28.6%), tonsillitis (11.7%, 95% CI 8.1%-15.3%), difficulty breathing (11.7%, 95% CI 8.1%-16.3%), pus or blood in stools (11.5%, 95% CI 10.7%-12.2%), tenesmus/constipation (10.7%, 95% CI 10.0%-11.5%), perianal ulcer (9.9%, 95% CI 4.3%-15.5%), and tenesmus/constipation (9.4%, 95% CI 6.4%-12.4%) were newly reported and had a lower prevalence. Other past reported symptoms, including dysphagia/difficulty swallowing (27.9%, 95% CI 21.1%-34.7%), scrotal edema (25.9%, 95% CI 0%-60.8%), body pain (22.6%, 95% CI 10.4%-34.7%), arthralgia (21%, 95% CI 0.8%-41.2%), proctalgia/diarrhea (16.7%, 95% CI 1.8%-25.6%), nausea/vomiting (13.3%, 95% CI 2.2%-24.5%), corneal opacity/photophobia (10.4%, 95% CI 0%-25.5%), nasal congestion/rhinorrhea (7.1%, 95% CI 0%-16.2%), and seizures (0.7%, 95% CI 0%-4.1%) remained steady, while the prevalence of sore throat (25.1%, 95% CI 20.4%-29.9%), mouth ulcers (13.7%, 95% CI 8.2%-19.2%), cough (8.1%, 95% CI 3.9%-12.3%), and conjunctivitis (5.4%, 95% CI 4.6%-6.2%) was lower than that in former periods.

**Figure 5:**
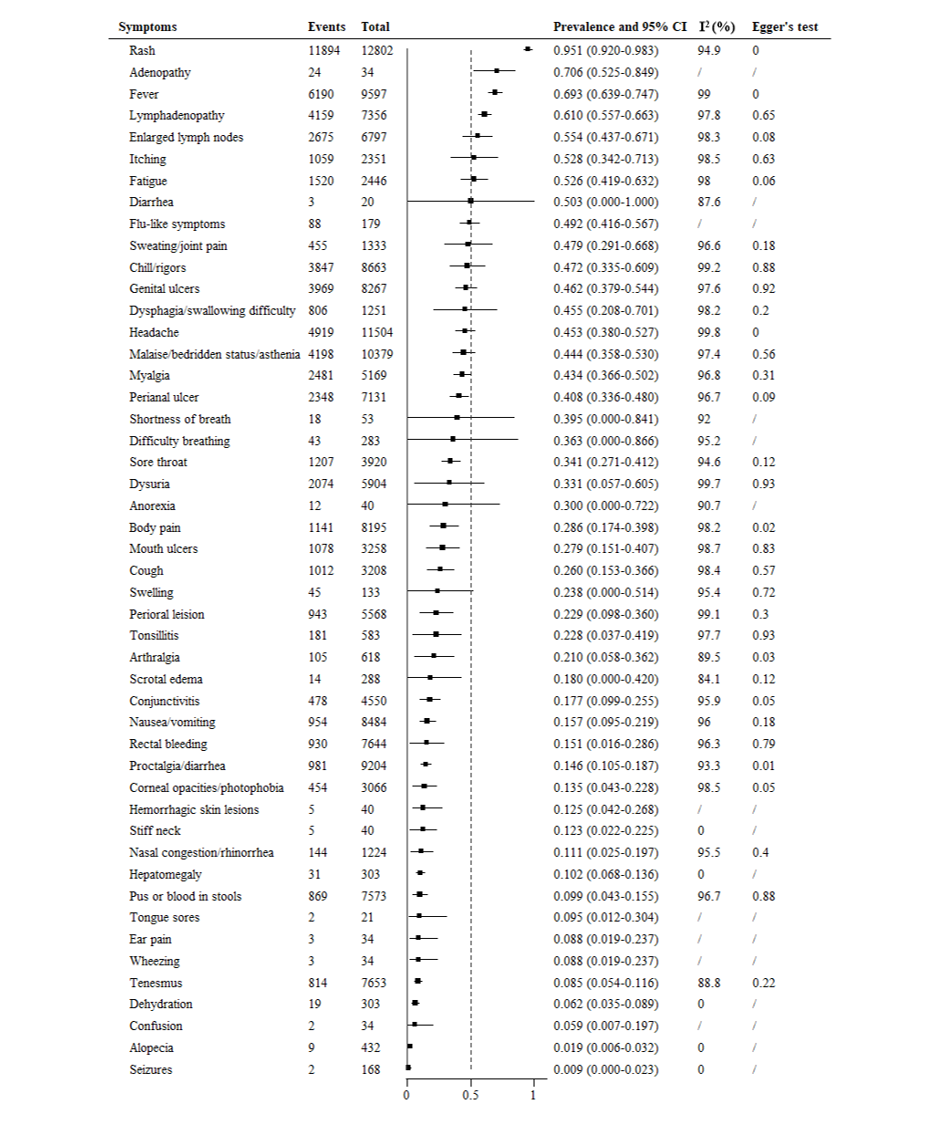
Meta-analysis of the prevalence of clinical symptoms among mpox patients from 1970 to 2023. mpox: monkeypox.

The prevalence of symptoms from 1970 to 2023 was included in the meta-analysis as well, involving 51 reported symptoms from 13,196 patients (Figure 5). Generally, rash (95.1%, 95% CI 92.0%-98.3%) was the prevalent symptom, followed by adenopathy (70.6%, 95% CI 52.5%-84.9%), fever (69.3%, 95% CI 63.9%-74.1%), and lymphadenopathy (61.0%, 95% CI 55.7%-66.3%), enlarged lymph nodes (55.4%, 95% CI 43.7%-67.1%), itching (52.8%, 95% CI 34.2%-71.3%), fatigue (52.6%, 95% CI 41.9%-63.2%), and diarrhea (50.3%, 95% CI 0%-100%). The prevalence of the other 40 (78.4%) symptoms was <50%. The prevalence of 14 (27.5%) symptoms ranged from 30% to 50%, including flu-like symptoms (49.2%, 95% CI 41.6%-46.7%), sweating/joint pain (47.9%, 95% CI 29.1%-66.8%), and chills/rigors (47.2%, 95% CI 33.5%-60.9%). For 17 (33.3%) symptoms, the prevalence ranged from 10% to 30%, including body pain (28.6%, 95% CI 17.4%-39.8%), mouth ulcers (27.9%, 95% CI 15.1%-40.7%), and cough (26%, 95% CI 15.3%-33.6%). Another 9 (17.6%) symptoms, such as tongue sores (9.5%, 95% CI 1.2%-30.4%), ear pain (8.8%, 95% CI 1.9%-23.7%), and tenesmus (8.5%, 95% CI 5.4%-11.6%), had <10% prevalence.

### Sensitivity Analysis

We excluded studies with moderate and higher risk of bias, including 3 (4.9%) studies from 1970 to 2002, 6 (9.8%) studies from 2003 to 2021, and 23 (37.7%) studies since 2022 (Table S2 in Multimedia Appendix 1). A total of 29 (47.5%) studies were included in the sensitivity analysis. In the 3 periods, rash (period 1: 100.00%, 95% CI 98.7%-100%; period 2: 99%, 95% CI 97.1%-100.00%; and period 3: 93.1%, 95% CI 87.7%-98.6%) was the most common symptom, followed by fever (period 1: 100.00%, 95% CI 98.7%-100%; period 2: 89.4%, 95% CI 83.1%-95.6%; and period 3: 58.3%, 95% CI 52.8%-63.7%). The other symptoms with a relatively higher prevalence included lymphadenopathy, headache, and fatigue. Significantly, symptom prevalence from 1970 to 2002 changed compared to former meta-analysis results, while that in the other 2 periods did not, and almost all symptom prevalence decreased since 2022 (Figure S2 in Multimedia Appendix 1).

### Subgroup Meta-Analysis

To compare the geographical differences in clinical symptoms, we performed a subgroup analysis of the symptom prevalence in 3 periods (2003-2021, 2022-2023, and 1970-2023) based on the WHO regional distribution. We did not conduct a subgroup analysis for the period from 1970 to 2002 since all patients were in the African region. From 2003 to 2021, 30 symptoms were reported in Africa, 26 in the Americas, 6 in Europe, and 10 in the Western Pacific. The overall prevalence of symptoms was highest in Africa, particularly in terms of rash (100%, 95% CI 99.9%-100%), fever (92%, 95% CI 85.7%-98.2%), dysphagia/difficulty swallowing (85.5%, 95% CI 57.4%-100%), chills/rigors (73.9%, 95% CI 55.3%-92.5%), headache (66.3%, 95% CI 52.9%-79.6%), shortness of breath (63.2%, 95% CI 38.4%-83.7%), and conjunctivitis (25.3%, 95% CI 17.3%-33.4%), which were significantly higher than in some areas (*P*≤.05). Furthermore, characteristic symptoms in African cases included difficulty breathing (63.2%, 95% CI 38.4%-83.7%), genital ulcers (57.1%, 95% CI 43.0%-71.1%), mouth ulcers (53.6%, 95% CI 33.0%-74.3%), anorexia (30%, 95% CI 0%-72.2%), corneal opacity/photophobia (27.1%, 95% CI 18.9%-35.3%], 78.7%), and hemorrhagic skin lesions (12.5%, 95% CI 4.2%-26.8%), whose prevalence was >10%. However, the prevalence of nausea/vomiting (59.7%, 95% CI 0%-100%) in cases from the Americas was higher than in Africa (*P*≤.05), and some characteristic symptoms, including adenopathy (70.6%, 95% CI 52.5%-74.9%) and stiff neck (11.8%, 95% CI 33.0%-27.5%), were reported exclusively in the Americas (Table S3 in Multimedia Appendix 1).

Since 2022, cases have been primarily concentrated in the Americas (27 symptoms) and Europe (25 symptoms), with fewer patients reported in the Western Pacific (12 symptoms) and Eastern Mediterranean (7 symptoms) and no cases reported in Africa. The prevalence of symptoms in the 4 regions had generally no significant difference. However, the risk of enlarged lymph nodes (61.3%, 95% CI 49.3%-73.2%) and rectal bleeding (60%, 95% CI 36.1%-80.9%) in European cases was higher than in the Americas (*P*≤.05). Eastern Mediterranean cases exhibited a higher prevalence of headache (100%, 95% CI 15.8%-100%) and fatigue (100%, 95% CI 15.8%-100%), but the number of cases was relatively low. Additionally, the characteristic symptom presentations in the Americas were sweating/joint pain (35.2%, 95% CI 32.4%-38.2%), nausea/vomiting (18.1%, 95% CI 3.5%-32.7%), pus or blood in stools (11.7%, 95% CI 6.2%-17.3%), and tenesmus/constipation (10.6%, 95% CI 8.4%-12.9%) and in Europe were flu-like symptoms (49.2%, 95% CI 41.6%-56.7%), scrotal edema (45.5%, 95% CI 24.7%-66.2%), arthralgia (32.4%, 95% CI 0%-75.6%), corneal opacity/photophobia (18.7%, 95% CI 0%-51.1%), tonsillitis (11.7%, 95% CI 8.1%-15.3%), and difficulty breathing (11.7%, 95% CI 8.1%-16.3%); see Table S4 in Multimedia Appendix 1.

The merged periods showed more differences in symptom prevalence among regions. Generally, rash was a prevalent symptom, with its prevalence reaching close to 100% in all regions. Africa had the highest prevalence of the most symptoms, such as fever (94.5%, 95% CI 89.9%-99.0%), headache (68%, 95% CI 51.7%-84.3%), and chills/rigors (73.9%, 95% CI 55.3%-92.5%; *P*≤.05), followed by the Americas and Europe, which showed similar risks for the main symptoms. Nonendemic regions, such as the Western Pacific and Eastern Mediterranean, reported fewer symptoms, and the prevalence of the main symptoms was similar to that in Europe and the Americas (Table S5 in Multimedia Appendix 1).

### Association Between Clinical Symptoms

**Figure 6:**
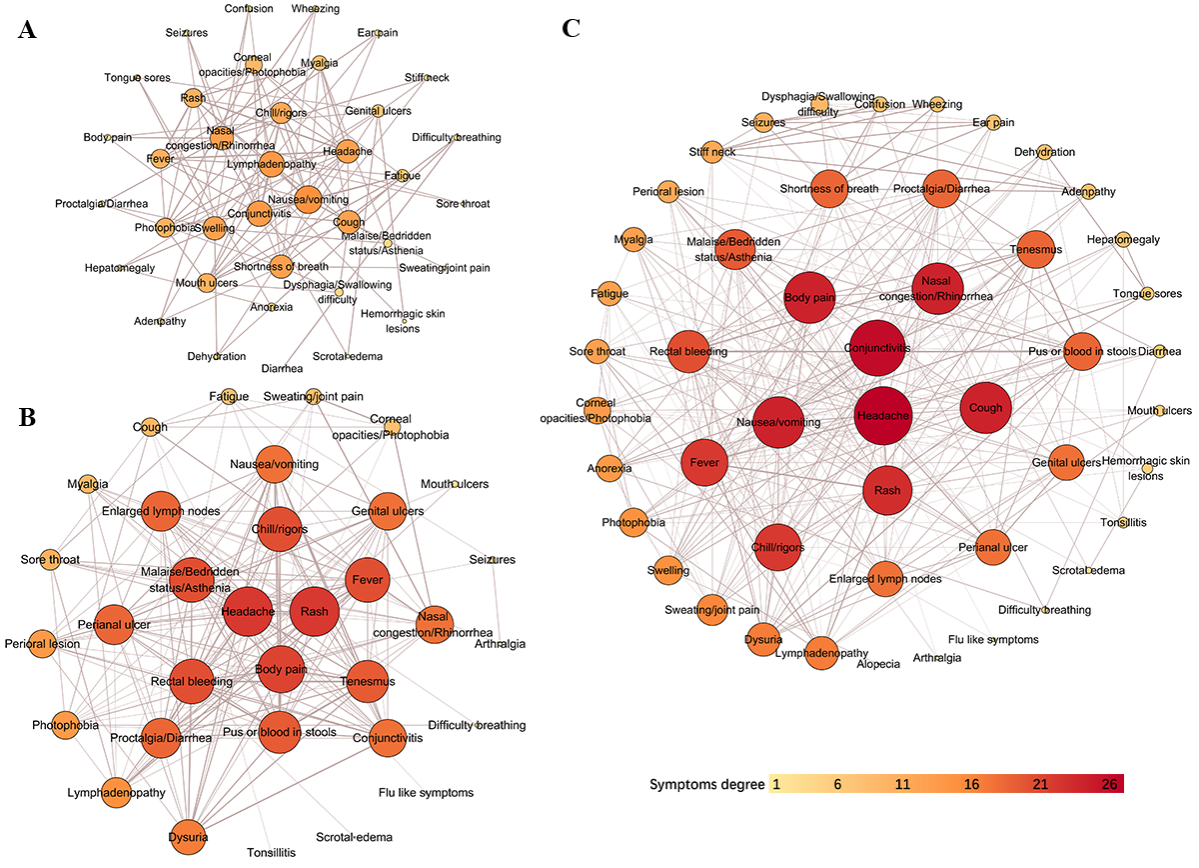
Network diagram of clinical symptoms among mpox patients in (A) 2003-2021, (B) 2022-2023, and (C) 1970-2023. mpox: monkeypox.

We conducted Pearson correlation analysis on the clinical symptoms, screening out significantly correlated paired symptoms (*P*≤.05) and drawing a network diagram of 3 periods: 2003-2021, 2022-2023, and the overall period of 1970-2023 (Figure 6a-c). From 1970 to 2002, only 14 symptoms were found due to insufficient literature data, so we did not draw a network diagram specifically for this period.

A total of 37 symptoms showed a significantly positive correlation from 2003 to 2021 (Figure 6a). Among them, nausea/vomiting was variable with the highest degree of correlation and was positive correlated with 13 symptoms: lymphadenopathy (r=0.908), conjunctivitis (r=0.900), cough (r=0.878), chills/rigors (r=0.791), headache (r=0.739), photophobia (r=0.720), fatigue (r=0.713), fever (r=0.693), corneal opacity (r=0.676), rash (r=0.652), mouth ulcers (r=0.648), malaise/bedridden status/asthenia (r=0.648), and myalgia (r=0.648). This was followed by lymphadenopathy (correlated with 12 symptoms), conjunctivitis (12 symptoms), nasal congestion/rhinorrhea (11 symptoms), swelling (11 symptoms), headache (11 symptoms), cough (11 symptoms), shortness of breath (11 symptoms), and chills/rigors (10 symptoms). A total of 11 symptoms (proctalgia/diarrhea, mouth ulcers, rash, fever, photophobia, stiff neck, ear pain, wheezing, confusion, seizures, and adenopathy) were correlated with 9 other symptoms. In addition, corneal opacity was correlated with 8 symptoms, myalgia with 7 symptoms, and fatigue and genital ulcers with 6 symptoms. Another 13 symptoms (hepatomegaly, dehydration, tongue sores, dysphagia/difficulty swallowing, malaise/bedridden status/asthenia, anorexia, body pain, scrotal edema, hemorrhagic skin lesions, difficulty breathing, sore throat, sweating/joint pain, and diarrhea) had no more than 5 correlated clinic characteristics.

Since 2022, a more complicated network diagram involving 33 symptoms illustrated a substantial positive correlation (Figure 6b). Generally, rash and headache had the highest degrees of correlation and were correlated with 21 symptoms. The symptom with the highest correlation with rash was fever (r=0.918), followed by lymphadenopathy (r=0.789), headache (r=0.787), body pain (r=0.610), malaise/bedridden status/asthenia (r=0.575), genital ulcers (r=0.558), proctalgia/diarrhea (r=0.554), pus or blood in stools (r=0.549), tenesmus (r=0.548), perianal ulcer (r=0.543), and rectal bleeding (r=0.508). In addition, fever and rash were also the most highly correlated with headache (r=0.789 and 0.788, respectively). Other symptoms with a high degree of correlation included body pain (20 symptoms), fever (19 symptoms), malaise/bedridden status/asthenia (19 symptoms), chills/rigors (19 symptoms), rectal bleeding (19 symptoms), pus or blood in stools (18 symptoms), tenesmus (18 symptoms), enlarged lymph nodes (17 symptoms), perianal ulcer (17 symptoms), and proctalgia/diarrhea (17 symptoms).

We also mapped the network diagram after merging 3 periods. The symptom with the highest number of associated symptoms (n=26) was headache (Figure 6c). Among them, the 2 symptoms with the highest correlation were fever (r=0.780) and rash (r=0.754), followed by lymphadenopathy (r=0.576), myalgia (r=0.507), and proctalgia/diarrhea (r=0.500); the r value for the other 21 symptoms was <0.500. Conjunctivitis had the second-highest degree of correlation and was correlated with 25 symptoms: it has high correlation with nausea/vomiting (r=0.844), and the correlation with the other 24 symptoms was relatively low (r<0.05). Other symptoms with a high degree of correlation included body pain (23 symptoms), nausea/vomiting (23 symptoms), cough (23 symptoms), nasal congestion/rhinorrhea (23 symptoms), rash (22 symptoms), fever (21 symptoms), chills/rigors (21 symptoms), rectal bleeding (19 symptoms), malaise/bedridden status/asthenia (18 symptoms), proctalgia/diarrhea (17 symptoms), pus or blood in stools (17 symptoms), shortness of breath (17 symptoms), and tenesmus (17 symptoms).

We extensively researched the data related to the mpox death toll reports between 1970 and 2023 from various literature sources and websites. The findings are presented in Table S6, Table S7, and Figure S8 in Multimedia Appendix 1. Initially, high case fatality rates (CFRs) were predominantly observed before 1986: CFR=16.7% in 1970, 60% in 1972, 33.3% in 1973, 16.7% in 1975, 23.1% in 1978, and 9.7%-12.5% from 1981 to 1985. No death cases were reported from 1987 to 1995. Subsequently, CFRs remained below 6% between 1996 and 2022. The highest CFR occurred in 2016 (5.7%), followed by 2002 (4.2%), 2008 (4.2%), and 2001 (3.8%). In 2022, the CFR of mpox decreased to 0.09% but increased to 1.85% in 2023. The countries with the highest CFRs in 2023 were Belgium (33.33%), Peru (7.64%), and the United States (4.45%); see Figure S3c in Multimedia Appendix 1. To investigate the relationship between symptoms and death, we included 6 (9.8%) papers reporting outcome indicators related to death. The network analysis showed that rash (r=0.841), nausea/vomiting (r=0.832), conjunctivitis (r=0.832), and corneal opacity/photophobia (r=0.912) were significantly associated with mortality (*P*≤.05); see Figure S4 in Multimedia Appendix 1.

## Discussion

### Principal Findings

The clinical symptoms of mpox have shown a decreasing prevalence over time, except for the specific symptom of rash, which has remained consistent. However, there has been an increasing concurrent prevalence of symptoms, resulting in a more complex correlation among them. The prevalence estimates of most symptoms were higher in Africa from 1970 to 2021, with about half of them occurring since 2003. The risks have shifted to Europe and the Americas since the 2022 outbreak, accompanied by increases in age and the proportions of males and MSM among the patients.

### General Evolution Trends

An important highlight is that the presence of rash remained consistent in confirmed cases of monkeypox across all periods studied. This characteristic rash plays a vital role in the accurate diagnosis of poxvirus infections, emphasizing its significance as a key determinant in distinguishing monkeypox from other similar conditions [27]. A consistent and noteworthy trend observed throughout various periods is the continuous reporting of key symptoms, such as fever, lymphadenopathy, headache, cough, and sore throat, in cases of mpox. Although some of these symptoms have displayed a higher prevalence compared to others, they have remained steady since 2003 and decreased since the onset of the 2022 pandemic, which is roughly consistent with the previous 2 meta-analyses with pre- and post-2022 studies [21,22]. Especially in the pandemic since 2022, the prevalence of coreported symptoms was similar to other meta-analyses in 2022 [17,18]. As all symptoms were included in our study, it was observed for the first time that the number of symptoms has increased since 2003 and has remained steady throughout the worldwide pandemic. The decrease in the prevalence of nonspecific symptoms indicated an increase in asymptomatic cases, while new symptoms emerged, revealing a complex correlation among symptoms due to the evolution of mpox influenced by various factors.

### Geographical Differences

The main reason for this evolution can be explained by differences in the geographical characteristics, population structure, virus source, transmission routes, and medical environment among the 3 periods. Before 2022, Africa emerged as the primary endemic region, exhibiting higher mpox prevalence and more reported symptoms. The epidemic trend can be divided into 2 periods, based on the characteristic patterns of symptom prevalence: before and after the virus spread to the Americas. In the first period (1970-2002), the patients with mpox all occurred in Midwest Africa, and children had the highest proportion among confirmed cases, which was because most of them had not been vaccinated against smallpox, resulting in a higher case prevalence. It was reported that severe symptoms of mpox tend to manifest more frequently in children rather than in adults [14]. As a consequence, fever, enlarged lymph nodes, and headache were reported with a higher prevalence and were closely associated with the risk of rash. Although mpox has been reported in non-African countries since 2003, the disease remains predominantly prevalent in central African nations. During this transitional period, there has been a gradual increase in the average age of patients and the proportion of male patients affected by monkeypox. Therefore, the polled prevalence of primary symptoms experienced a mild decrease from 2003 to 2021. However, the African patients still remained at higher risk of main symptoms and reported new symptoms, compared to nonepidemic regions, such as Europe and the Western Pacific, while most nonepidemic regions cases were imported from Africa. As the first outbreak outside Africa, cases in the Americas were mostly confirmed in 2003, with relatively higher reported symptom prevalence. Since the 2022 outbreak, Europe and the Americas emerged as 2 epidemic regions that reported a majority of diagnosed cases, with predominantly adult patients, resulting in a relatively favorable clinical presentation in patients. The prevalence of coreported symptoms exhibited consistent characteristics thorough the epidemic and nonepidemic regions. Additionally, a higher number of confirmed cases and more comprehensive treatment modalities in endemic regions led to more than half of the newly reported symptoms, while the prevalence was generally lower.

### Clade Evolution

The varying clades of the mpox virus could potentially exert a significant influence on symptom severities. Two major clades, the Congo Basin clade (clade I) and the West African clade (clade II), evolved from Africa and exhibited a potential impact on the clinical characteristics of patients with mpox. The Congo Basin clade was reported from 1970 to 2018 and is associated with severe disease and higher mortality [28]. The West African clade is associated with mild lower mortality and is divided into clade IIa and clade IIb [15,28,29], the former being reported only in the west of the Dahomey Gap from 1962 to 1971 and the United States in 2003 and the latter being reported in Nigeria from 1971 to 1978 and from 2017 to 2019 and worldwide since the 2022 outbreak [30]. Therefore, although the majority of the literature we included did not report branch data, it can be confirmed that a contributing factor to the significantly reduced severity of symptoms in 2022 was the lower virulence of clade IIb compared with clade I [31]. However, concerning the period before 2022, the impact of clades is rather intricate. For the African cases between 2003 and 2021, the prevalence remained unchanged or increased compared to pre-2003, while the prevalence of symptoms in other regions during the same period was relatively lower. For instance, the mpox outbreak in the United States in 2003 was caused only by clade IIa. Despite having a similar clinical characteristic to Africa, the prevalence of symptoms such as chills/rigors, dysphagia/difficulty swallowing, conjunctivitis, and shortness of breath was significantly lower. In addition, the disease has transitioned from being primarily animal-to-human transmitted to being human-to-human transmitted [10]. Animal-to-human transmission mostly occurred in 1970-2002. In nonendemic countries [13], few cases have been reported that were linked to international travel or the importation of animal-to-human monkeypox infection from 2003 to 2021 [32]. Mpox infection at this stage was not attributed to person-to-person contact [33]. In 2022, the majority of cases, with a few exceptions, were identified following instances of human-to-human sexual contact [34]. Reduction in symptom severities may depend on the viral clade [33] to better adapt to human-to-human transmission. Furthermore, favorable living environments and advanced medical conditions in high-income countries or regions may also explain the decrease in symptom prevalence since the 2022 outbreak.

### Correlation Among Symptoms

Although the escalation in the number of comorbidities resulted in more intricate interconnections between symptoms, which mutually reinforced the correlation between them in our network diagram. This can be explained by more detailed symptom reports, which contribute to enhanced monitoring and advanced medical conditions. More importantly, a fact is that all symptoms are positively correlated, indicating that they are mutually reinforcing. Furthermore, the human-to-human transmission route has changed. Although HIV-related complications were noted during the monkeypox outbreak in Nigeria from 2017 to 2018, no substantiated evidence exists to indicate that the patients engaged in MSM behavior [35]. Subsequent to the outbreak in 2022, the studies incorporated in our analysis have consistently revealed a predominance of male patients, a substantial proportion of whom reported engaging in MSM behavior. These individuals also presented with coinfection, including HIV, syphilis, and various other STIs. Therefore, the mpox virus transmitted through MSM behavior has caused a rise in the number of additional symptoms, particularly an increase in the prevalence of symptoms associated with MSM behavior, such as perianal ulcer, pus or blood in stools, rectal bleeding, tenesmus/constipation, and dysuria. This may lead to complex response measures and treatment modalities.

### Risks Factors of CFRs

All evidence suggests that the mpox virus is becoming mild, including a decrease in hospitalization rates in the reported literature [36] and the CFR in our collected data. The CFR is a complex outcome and not only is influenced by differences in symptoms caused by mpox clades but also is significantly correlated with patients’ age and local economic conditions [37]. This is one of the reasons why the CFR was higher before 2003. However, the CFR rebounded in 2023. This may be due to the lag in reporting death data compared to confirmed reports. However, the STI coinfection caused by sexual contact may have a synergistic effect with the mpox virus, increasing the severity of the disease.

### Implications

Our findings are crucial for a comprehensive understanding of the mpox epidemic and for guiding an adequate policy from an epidemiological perspective. First, surveilling the evolving nature of mpox and the consequential changes in clinical characteristics is advised, especially in the epidemic regions, where the policy makers may shift their focus on the potential association between symptoms and the synergy risks to mortality. Furthermore, mpox can be influenced by a range of environmental and human factors. If appropriate measures are not taken to mitigate these factors, there is a heightened likelihood of a resurgence of the epidemic. Therefore, it is crucial to maintain ongoing vigilance and surveillance efforts, even in areas or periods when the disease appears to have been eliminated, in order to rapidly detect and respond to outbreaks and prevent the spread of the disease. However, targeted vaccination programs can be implemented for high-risk populations, including health care workers, laboratory workers, and those working with animals. Finally, efforts to raise public awareness, particularly within high-risk populations, such as MSM, should also be prioritized to help ensure timely detection and response to any potential outbreaks.

### Limitations and Strengths

This systematic review and meta-analysis has several limitations. First, the clinical symptoms were only collected from available data, so the results may fail to represent all patients with mpox, especially those in low- and middle-income countries in early studies. The included studies from 1970 to 2002 were limited and of low quality. Especially after 1986, WHO terminated the surveillance program for mpox, which may have underestimated the severity of the symptoms in the results. Second, the symptoms reported during 3 study periods were likely to have bias due to the varying study designs of each year. In addition, the different strains of mpox and transmission routes may have had a direct relevance for how clinically severe the illness was during the study period, which requires further genome-wide analysis to confirm it. In future analyses, it is important to consider other sources of heterogeneity that were not fully documented before the 2022 outbreak, such as age, case determination, and comorbidities. Finally, we cannot confirm which symptoms pose a higher risk of mortality during the worldwide outbreak since 2022, as limited data have simultaneously reported both the clinical presentation and the outcome of death; therefore, further evidence is needed to interpret this correlation.

Despite this, our review has carefully considered the changing trend of mpox symptoms since the first diagnosis in 1970 and also collected data on the number of confirmed cases of and deaths due to monkeypox in various countries over the years. It provided the evolution tendency of mpox, which can be used for making a related health policy. For example, we may improve treatment targeting for the common symptoms of mpox; according to demographic characteristics, we could suggest building an effective surveillance system for high-risk populations, especially in high-prevalence regions.

### Conclusion

During the worldwide mpox outbreak, the clinical symptoms, including rash, resembled what they had been prior to the outbreak; however, some unique symptoms and changes in transmission route should be noted. The exponential increase in positive cases in Europe and the United States may help build relevant early-warning systems to prevent more mpox cases.

## Appendix

Multimedia Appendix 1 Supplementary files for publication.

## Abbreviations

CFR: case fatality rate
DRC: Democratic Republic of the Congo
mpox: monkeypox
MSM: men who have sex with men
PRISMA: Preferred Reporting Items for Systematic Reviews and Meta-Analyses
STI: sexually transmitted infection
WHO: World Health Organization
