# Supplementary material for: Integrated Network Analysis of Symptom Clusters Across Monkeypox Epidemics From 1970 to 2023: Systematic Review and Meta-Analysis
Source: JMIR Public Health Surveill. 2024 Feb 16;10:e49285. doi: 10.2196/49285 (PMC10907939; doi:10.2196/49285)
Supplement: Multimedia Appendix 1 [file publichealth_v10i1e49285_app1.docx]

**Appendix**

**Table S1 Search strategies per database**

| Database | Search Term |
| --- | --- |
| PubMed | ((monkeypox) OR (monkeypox virus) OR (human monkeypox)) AND ((symptoms) OR (characteristics) OR (clinical characteristics) OR (clinical symptoms) OR (cases) OR (cases report)) AND ("1970/01/01"[Date - Publication]: "2023/04/30"[Date - Publication]) NOT (review) |
| Web of Science | TS=(((monkeypox) OR (monkeypox virus) OR (human monkeypox)) AND ((symptoms) OR (characteristics) OR (clinical characteristics) OR (clinical symptoms) OR (cases) OR (cases report)) NOT (review))  Index Date 1970-01-01 to 2023-04-30 |
| ScienceDirect | (monkeypox) AND ((symptoms) OR (characteristics) OR (clinical characteristics) OR (clinical symptoms) OR (cases) OR (cases report)) NOT (review)  Years 1970-2023 |

**Table S2 Risk of bias assessment with ROBINS-I tool**

| Study | Bias due to confounding | Bias in selection of participants into the study | Bias in classification of interventions | Bias due to deviations from intended interventions | Bias due to missing data | Bias in measurement of outcomes | Bias in selection of the reported result | Overall bias |
| --- | --- | --- | --- | --- | --- | --- | --- | --- |
| Foster et al (1972) [1] | Serious | NI | NI | NI | Low | NI | Low | Serious |
| Jezek et al (1987) [2] | Low | Low | NI | Low | Low | Low | Low | Low |
| Pebody et al (1997) [3] | Moderate | NI | NI | Low | Low | NI | Low | Moderate |
| Hutin et al (2001) [4] | Serious | Low | NI | Low | Moderate | NI | Low | Critical |
| Huhn et al (2005) [5] | Low | Low | NI | Low | Low | Low | Low | Low |
| Formenty et al (2010) [6] | Moderate | Low | NI | Low | Low | NI | Low | Moderate |
| Whitehouse et al (2021) [7] | Low | Low | NI | Low | Low | Low | Low | Low |
| Singapore Zika Study Group (2017) [8] | Low | Low | NI | Low | Low | Low | Low | Low |
| Yinka-Ogunleye et al (2019) [9] | Low | Low | NI | Low | Low | Low | Low | Low |
| Ogoina et al (2019) [10] | Low | Low | NI | Low | Low | Low | Low | Low |
| Ogoina et al (2020) [11] | Low | Low | NI | Low | Low | Low | Low | Low |
| Vaughan et al (2018) [12] | Moderate | Low | NI | Moderate | Serious | NI | Low | Critical |
| Yong et al (2020) [13] | Moderate | Low | NI | Low | Low | NI | Low | Moderate |
| Tumewu et al (2020) [14] | Moderate | Low | NI | Low | Low | NI | Low | Moderate |
| Hobson et al (2021) [15] | Moderate | Low | NI | Low | Serious | NI | Low | Critical |
| Rao et al (2022) [16] | Moderate | Low | NI | Low | Serious | NI | Low | Critical |
| Català et al (2022) [17] | Low | Low | NI | Low | Low | Low | Low | Low |
| Cassir et al (2022) [18] | Low | Low | NI | Low | Low | Low | Low | Low |
| de Sousa et al (2022) [19] | Low | Low | NI | Low | Low | Low | Low | Low |
| Girometti et al (2022) [20] | Low | Low | NI | Low | Low | Low | Low | Low |
| Martins-Filho et al (2022) [21] | Moderate | Low | NI | Low | Low | Low | Low | Moderate |
| Patel et al (2022) [22] | Low | Low | NI | Low | Low | Low | Low | Low |
| Philpott et al (2022) [23] | Low | Low | NI | Low | Low | Low | Low | Low |
| Thornhill et al (2022a) [24] | Serious | Low | NI | Low | Low | Low | Low | Serious |
| Tarín-Vicente et al (2022) [25] | Low | Low | NI | Low | Low | Low | Low | Low |
| Suñer et al (2022) [26] | Low | Low | NI | Low | Low | Low | Low | Low |
| Caria et al (2022) [27] | Low | Low | NI | Low | Low | Low | Low | Low |
| Simon F Roy et al (2023) [28] | Moderate | Low | NI | Low | Low | NI | Low | Moderate |
| Sihuincha et al (2022) [29] | Low | Low | NI | Low | Low | Low | Low | Low |
| Prasad et al (2023) [30] | Serious | Low | NI | Low | Low | Low | Low | Serious |
| Martínez et al (2022) [31] | Moderate | Low | NI | Low | Low | NI | Low | Moderate |
| Patalon et al (2022) [32] | Moderate | Low | NI | Low | Low | NI | Low | Moderate |
| Proietti et al (2022) [33] | Moderate | Low | NI | Low | Low | NI | Low | Moderate |
| Manoharan et al (2022) [34] | Moderate | Low | NI | Low | Low | NI | Low | Moderate |
| Pisano et al (2022) [35] | Moderate | Low | NI | Low | Low | NI | Low | Moderate |
| Cutoiu et al (2022) [36] | Moderate | Low | NI | Low | Low | NI | Low | Moderate |
| Desgranges et al (2023) [37] | Moderate | Low | NI | Low | Low | NI | Low | Moderate |
| Angelo et al (2023) [38] | Low | Low | NI | Low | Low | Low | Low | Low |
| Zayat et al (2023) [39] | Moderate | Low | NI | Low | Low | NI | Low | Moderate |
| Berens-Riha et al (2022) [40] | Moderate | Low | NI | Low | Low | NI | Low | Moderate |
| Oakley et al (2022) [41] | Low | Low | NI | Low | Low | Low | Low | Low |
| Farrar et al (2022) [42] | Low | Low | NI | Low | Low | Low | Low | Low |
| Kyaw et al (2022) [43] | Low | Low | NI | Low | Low | Low | Low | Low |
| Assiri et al (2023) [44] | Low | Low | NI | Low | Low | Low | Low | Low |
| Palich et al (2023) [45] | Low | Low | NI | Low | Low | Low | Low | Low |
| Alpalhão et al (2023) [46] | Moderate | Low | NI | Low | Low | Low | Low | Moderate |
| Rimmer et al (2023) [47] | Low | Low | NI | Low | Low | Low | Low | Low |
| Jang et al (2023) [48] | Moderate | Low | NI | Low | Low | NI | Low | Moderate |
| Huang et al (2023) [49] | Moderate | Low | NI | Low | Low | NI | Low | Moderate |
| Vallée et al (2023) [50] | Moderate | Low | NI | Low | Low | NI | Low | Moderate |
| Hennessee et al (2022) [51] | Low | Low | NI | Low | Low | NI | Low | Low |
| Thornhill et al (2022b) [52] | Low | Low | NI | Low | Low | NI | Low | Low |
| Choudhury et al (2022) [53] | Moderate | Low | NI | Low | Low | Low | Low | Moderate |
| Pipitò et al (2022) [54] | Moderate | Low | NI | Low | Low | NI | Low | Moderate |
| Lopes et al (2023) [55] | Serious | Low | NI | Serious | Low | NI | Low | Critical |
| Mailhe et al (2023) [56] | Low | Low | NI | Low | Low | Low | Low | Low |
| Noe et al (2023) [57] | Moderate | Low | NI | Low | Low | Low | Low | Moderate |
| Nouchi et al (2023) [58] | Low | Low | NI | Low | Low | Low | Low | Low |
| Srichawla et al (2023) [59] | Moderate | Low | NI | Low | Low | Low | Low | Moderate |
| García-Piqueras et al (2023) [60] | Serious | Low | NI | Low | Low | Low | Low | Serious |
| Rekik et al (2023) [61] | Low | Low | NI | Low | Low | Low | Low | Low |

NI, no information.

**Table S3 Subgroup meta-analysis of the prevalence of clinical symptoms among mpox patients from 2003 to 2021**

| Symptoms | Africa | | | | Americas | | | | Europe | | | | Western Pacific | | | | *P* |
| --- | --- | --- | --- | --- | --- | --- | --- | --- | --- | --- | --- | --- | --- | --- | --- | --- | --- |
|  | Events | Total | Prevalence and 95 CI | I2 (%) | Events | Total | Prevalence and 95 CI | I2 (%) | Events | Total | Prevalence and 95 CI | I2 (%) | Events | Total | Prevalence and 95 CI | I2 (%) |  |
| Rash | 1259 | 1259 | 1.000 (0.999-1.000) | 0 | 34 | 35 | 0.971 (0.904-1.000) | 0 | 4 | 5 | 0.852 (0.527-1.000) | 5.6 | 155 | 165 | 0.939 (0.902-0.976) | 0 | *< .01* |
| Fever | 1201 | 1231 | 0.920 (0.857-0.982) | 83.6 | 30 | 35 | 0.859 (0.740-0.977) | 0 | 2 | 5 | 0.395 (0.000-0.818) | 0 | 133 | 165 | 0.808 (0.747-0.868) | 0 | *.01* |
| Headache | 932 | 1213 | 0.663 (0.529-0.796) | 81.7 | 22 | 34 | 0.647 (0.465-0.803) | / | 1 | 2 | 0.500 (0.013-0.987) | / | 38 | 163 | 0.233 (0.171-0.306) | / | *< .01* |
| Malaise/bedridden status/asthenia | 291 | 1042 | 0.430 (0.091-0.768) | 90.5 | 4 | 34 | 0.118 (0.033-0.275) | / | / | / | / | / | / | / | / | / | *.09* |
| Genital ulcers | 35 | 61 | 0.571 (0.430-0.711) | 19.9 | / | / | / | / | / | / | / | / | / | / | / | / | */* |
| Lymphadenopathy | 1023 | 1236 | 0.781 (0.691-0.871) | 78.2 | 19 | 34 | 0.559 (0.379-0.728) | / | 1 | 2 | 0.500 (0.013-0.987) | / | / | / | / | / | *.06* |
| Chill/rigors | 930 | 1149 | 0.739 (0.553-0.925) | 94.4 | 24 | 34 | 0.706 (0.525-0.849) | / | / | / | / | / | / | / | / | / | *< .01* |
| Myalgia | 835 | 1146 | 0.549 (0.256-0.842) | 94.5 | 19 | 34 | 0.559 (0.379-0.728) | / | / | / | / | / | 67 | 163 | 0.411 (0.335-0.491) | / | *.22* |
| Fatigue | 974 | 1191 | 0.662 (0.488-0.836) | 95.6 | 1 | 1 | 1.000 (0.025-1.000) | / | / | / | / | / | / | / | / | / | *.29* |
| Body pain | 36 | 80 | 0.400 (0.160-0.639) | 83.2 | 18 | 34 | 0.529 (0.351-0.702) | / | / | / | / | / | / | / | / | / | *.38* |
| Sore throat | 98 | 183 | 0.513 (0.406-0.621) | 38.4 | 21 | 34 | 0.618 (0.436-0.778) | / | 1 | 2 | 0.500 (0.013-0.987) | / | / | / | / | / | *.57* |
| Nausea/vomiting | 285 | 1212 | 0.170 (0.096-0.244) | 79.8 | 12 | 35 | 0.597 (0.000-1.000) | 78.1 | / | / | / | / | / | / | / | / | *< .01* |
| Mouth ulcers | 627 | 1159 | 0.536 (0.330-0.743) | 91.7 | / | / | / | / | / | / | / | / | / | / | / | / | */* |
| Itching |  | 1195 | 0.595 (0.449-0.742) | 84.7 | / | / | / | / | 1 | 2 | 0.500 (0.013-0.987) | / | / | / | / | / | *.79* |
| Cough | 608 | 1186 | 0.384 (0.206-0.561) | 94.6 | 17 | 35 | 0.653 (0.160-1.000) | 64 | / | / | / | / | / | / | / | / | *.31* |
| Proctalgia/diarrhea | / | / | / | / | 2 | 34 | 0.059 (0.007-0.197) | / | / | / | / | / | / | / | / | / | */* |
| Dysphagia/swallowing | 755 | 1051 | 0.855 (0.574-1.000) | 98.3 | 4 | 34 | 0.118 (0.033-0.275) | / | / | / | / | / | / | / | / | / | *< .01* |
| Conjunctivities | 264 | 1218 | 0.253 (0.173-0.334) | 65.2 | 3 | 34 | 0.088 (0.019-0.237) | / | / | / | / | / | / | / | / | / | *< .01* |
| Corneal opacities/photophobia | 368 | 1161 | 0.271 (0.189-0.353) | 78.7 | / | / | / | / | / | / | / | / | / | / | / | / | */* |
| Sweating/joint pain | 93 | 141 | 0..692 (0.551-0.832) | / | 17 | 34 | 0.500 (0.324-0.676) | / | / | / | / | / | / | / | / | / | *.09* |
| Nasal congestion/rhinorrhea | 5 | 40 | 0.125 (0.042-0.268) | / | 10 | 34 | 0.294 (0.151-0.475) | / | / | / | / | / | / | / | / | / | *.07* |
| Arthralgia | / | / | / | / | / | / | / | / | / | / | / | / | 36 | 163 | 0.221 (0.160-0.292) | / | */* |
| Swelling | 3 | 21 | 0.143 (0.030-0.363) | / | 2 | 34 | 0.059 (0.007-0.197) | / | / | / | / | / | / | / | / | / | *.33* |
| Difficulty breathing | 12 | 19 | 0.632 (0.384-0.837) | / | / | / | / | / | / | / | / | / | / | / | / | / | */* |
| Hepatomegaly | 2 | 21 | 0.095 (0.012-0.304) | / | / | / | / | / | / | / | / | / | / | / | / | / | */* |
| Adenopathy | / | / | / | / | 24 | 34 | 0.706 (0.525-0.749) | / | / | / | / | / | / | / | / | / | */* |
| Dehydration | 2 | 21 | 0.095 (0.012-0.304) | / | / | / | / | / | / | / | / | / | / | / | / | / | */* |
| Short of breath | 12 | 19 | 0.632 (0.384-0.837) | / | 6 | 34 | 0.176 (0.068-0.345) | / | / | / | / | / | / | / | / | / | *< .01* |
| Scrotal edema | 2 | 40 | 0.050 (0.006-0.169) | / | / | / | / | / | / | / | / | / | / | / | / | / | */* |
| Anorexia | 12 | 40 | 0.300 (0.000-0.722) | 90.7 | / | / | / | / | / | / | / | / | / | / | / | / | */* |
| Stiff neck | / | / | / | / | 4 | 34 | 0.118 (0.033-0.275) | / | / | / | / | / | / | / | / | / | */* |
| Hemorrhagic skin lesions | 5 | 40 | 0.125 (0.042-0.268) | / | / | / | / | / | / | / | / | / | / | / | / | / | */* |
| Ear pain | / | / | / | / | 3 | 34 | 0.088 (0.019-0.237) | / | / | / | / | / | / | / | / | / | */* |
| Wheezing | / | / | / | / | 3 | 34 | 0.088 (0.019-0.237) | / | / | / | / | / | / | / | / | / | */* |
| Diarrhea | 2 | 19 | 0.105 (0.013-0.331) | / | 1 | 1 | 1.000 (0.025-1.000) | / | / | / | / | / | / | / | / | / | *< .01* |
| Confusion | / | / | / | / | 2 | 34 | 0.059 (0.007-0.197) | / | / | / | / | / | / | / | / | / | / |
| Seizures | / | / | / | / | 1 | 34 | 0.029 (0.001-0.153) | / | / | / | / | / | / | / | / | / | / |
| Tongue sores | 2 | 21 | 0.095 (0.012-0.304) | / | / | / | / |  | / | / | / | / | / | / | / | / | / |

The use of the symbol "/" in the "Event," "Total," and "Prevalence and 95 CI" columns indicates that no data was reported. In the "I2" column, it means that heterogeneity cannot be calculated because there was only one included study. In the "P" column, it means that the difference cannot be calculated because there was only one subgroup.

**Table S4 Subgroup meta-analysis of the prevalence of clinical symptoms among mpox patients from 2022 to 2023**

| Symptoms | Americas | | | | Eastern Mediterranean | | | | Europe | | | | Western Pacific | | | | *P* |
| --- | --- | --- | --- | --- | --- | --- | --- | --- | --- | --- | --- | --- | --- | --- | --- | --- | --- |
|  | Events | Total | Prevalence and 95 CI | I2 (%) | Events | Total | Prevalence and 95 CI | I2 (%) | Events | Total | Prevalence and 95 CI | I2 (%) | Events | Total | Prevalence and 95 CI | I2 (%) |  |
| Rash | 7436 | 7948 | 0.951 (0.898-1.000) | 97.8 | 9 | 9 | 1.000 (0.844-1.000) | 0 | 1516 | 1675 | 0.957 (0.902-1.000) | 92.2 | 10 | 10 | 1.000 (0.868-1.000) | / | *.87* |
| Fever | 2043 | 3980 | 0.620 (0.478-0.762) | 92.4 | 7 | 9 | 0.813 (0.571-1.000) | 0 | 455 | 802 | 0.617 (0.526-0.708) | 73.9 | 6 | 10 | 0.700 (0.292-1.000) | 40 | *0.5* |
| Headache | 2973 | 7605 | 0.472 (0.382-0.561) | 95.8 | 2 | 2 | 1.000 (0.158-1.000) | / | 428 | 1203 | 0.366 (0.294-0.438) | 82.3 | 3 | 9 | 0.333 (0.075-0.701) | / | *.01* |
| Malaise/bedridden status/asthenia | 3387 | 7743 | 0.518 (0.401-0.635) | 94.5 | 2 | 2 | 1.000 (0.158-1.000) | / | 152 | 337 | 0.451 (0.284-0.617) | 82.4 | / | / | / | / | *.06* |
| Genital ulcers | 2668 | 6160 | 0.398 (0.331-0.465) | 95.6 | 3 | 7 | 0.429 (0.099-0.816) | / | 407 | 902 | 0.496 (0.381-0.602) | 94.7 | / | / | / | / | *.35* |
| Lymphadenopathy | 1112 | 2714 | 0.559 (0.370-0.758) | 98.1 | 7 | 9 | 0.823 (0.551-1.000) | 10.4 | 1012 | 1670 | 0.619 (0.551-0.688) | 90.6 | / | / | / | / | *.28* |
| Chill/rigors | 2820 | 6942 | 0.453 (0.355-0.551) | 96.6 | 1 | 2 | 0.500 (0.013-0.987) | / | / | / | / | / | 1 | 9 | 0.111 (0.003-0.482) | / | *.01* |
| Enlarged lymph nodes | 2040 | 5731 | 0.380 (0.316-0.443) | 81.7 | / | / | / | / | 326 | 558 | 0.613 (0.493-0.732) | 88.8 | / | / | / | / | *< .01* |
| Myalgia | 979 | 1959 | 0.491 (0.427-0.555) | 77.4 | 1 | 2 | 0.500 (0.013-0.987) | / | 324 | 872 | 0.435 (0.342-0.529) | 81.1 | 3 | 10 | 0.563 (0.000-1.000) | 81.4 | *.80* |
| Perianal ulcer | 1920 | 6159 | 0.359 (0.224-0.494) | 98.5 | / | / | / | / | 232 | 614 | 0.385 (0.316-0.453) | 57.2 | / | / | / | / | *.74* |
| Dysuria | 2069 | 5676 | 0.459 (0.249-0.668) | 98 | 1 | 2 | 0.500 (0.013-0.987) | / | / | / | / | / | / | / | / | / | *.91* |
| Fatigue | 105 | 205 | 0.512 (0.442-0.582) | / | 2 | 2 | 1.000 (0.158-1.000) | / | 24 | 56 | 0.429 (0.299-0.558) | 0 | 1 | 9 | 0.111 (0.003-0.482) | / | *< .01* |
| Body pain | 982 | 7489 | 0.238 (0.090-0.386) | 99.2 | / | / | / | / | 95 | 366 | 0.293 (0.000-0.607) | 97.8 | / | / | / | / | *.76* |
| Sore throat | 254 | 927 | 0.333 (0.189-0.477) | 88.2 | / | / | / | / | 244 | 1144 | 0.244 (0.165-0.323) | 86.7 | 2 | 10 | 0.510 (0.000-1.000) | 86.5 | *.49* |
| Nausea/vomiting | 647 | 6729 | 0.181 (0.035-0.327) | 92.9 | / | / | / | / | / | / | / | / | / | / | / | / | */* |
| Mouth ulcers | 179 | 1007 | 0.178 (0.155-0.203) | / | 1 | 7 | 0.143 (0.004-0.579) | / | 77 | 455 | 0.149 (0.046-0.252) | 89.2 | / | / | / | / | *.84* |
| Itching | 319 | 925 | 0.426 (0.013-0.838) | 97.7 | 2 | 2 | 1.000 (0.158-1.000) | / | 1 | 1 | 1.000 (0.025-1.000) | / | / | / | / | / | *.11* |
| Perioral | 845 | 5069 | 0.332 (0.000-0.797) | 99.8 | / | / | / | / | 81 | 370 | 0.196 (0.092-0.300) | 87.5 | / | / | / | / | *.58* |
| Cough | 126 | 925 | 0.320 (0.000-0.822) | 96.4 | / | / | / | / | 8 | 122 | 0.060 (0.018-0.102) | 0 | / | / | / | / | *.31* |
| Proctalgia/diarrhea | 725 | 7360 | 0.136 (0.073-0.199) | 97 | / | / | / | / | 77 | 329 | 0.227 (0.090-0.365) | 89.5 | / | / | / | / | *.24* |
| Rectal bleeding | 910 | 7398 | 0.102 (0.053-0.150) | 96.1 | / | / | / | / | 12 | 20 | 0.600 (0.361-0.809) | / | / | / | / | / | *< .01* |
| Pus or blood in stools | 863 | 7347 | 0.117 (0.062-0.173) | 95.4 | / | / | / | / | / | / | / | / | / | / | / | / | */* |
| Tenesmus/constipation | 801 | 7339 | 0.106 (0.084-0.129) | 75.2 | / | / | / | / | / | / | / | / | / | / | / | / | */* |
| Dysphagia/swallowing | / | / | / | / | / | / | / | / | 47 | 166 | 0.279 (0.211-0.347) | 0 | / | / | / | / | */* |
| Conjunctivities | 164 | 3016 | 0.054 (0.046-0.062) | 0 | / | / | / | / | / | / | / | / | / | / | / | / | */* |
| Corneal opacities/photophobia | 38 | 719 | 0.053 (0.038-0.072) | / | / | / | / | / | 32 | 334 | 0.187 (0.000-0.511) | 96.9 | / | / | / | / | *.42* |
| Sweating/joint pain | 324 | 926 | 0.352 (0.324-0.382) | / | / | / | / | / | / | / | / | / | / | / | / | / | */* |
| Tonsillitis | / | / | / | / | / | / | / | / | 36 | 301 | 0.117 (0.081-0.153) | 0 | / | / | / | / | */* |
| Nasal congestion/rhinorrhea | 124 | 924 | 0.096 (0.000-0.229) | 98.2 | / | / | / | / | / | / | / | / | / | / | / | / | */* |
| Arthralgia | / | / | / | / | / | / | / | / | 44 | 227 | 0.324 (0.000-0.750) | 96.6 | / | / | / | / | */* |
| Flu-like symptoms | / | / | / | / | / | / | / | / | 88 | 179 | 0.492 (0.416-0.567) | | / | / | / | / | */* |
| Difficulty breathing | / | / | / | / | / | / | / | / | 31 | 264 | 0.117 (0.081-0.163) | | / | / | / | / | */* |
| Scrotal edema | / | / | / | / | / | / | / | / | 10 | 22 | 0.455 (0.247-0.662) | 0 | / | / | / | / | */* |

The use of the symbol "/" in the "Event," "Total," and "Prevalence and 95 CI" columns indicates that no data was reported. In the "I2" column, it means that heterogeneity cannot be calculated because there was only one included study. In the "P" column, it means that the difference cannot be calculated because there was only one subgroup.

**Table S5 Subgroup meta-analysis of the prevalence of clinical symptoms among mpox patients from 1970 to 2023**

| Symptoms | Africa | | | | Americas | | | | Eastern Mediterranean | | | | Europe | | | | Western Pacific | | | | *P* |
| --- | --- | --- | --- | --- | --- | --- | --- | --- | --- | --- | --- | --- | --- | --- | --- | --- | --- | --- | --- | --- | --- |
|  | Events | Total | Prevalence and 95 CI | I2 (%) | Events | Total | Prevalence and 95 CI | I2 (%) | Events | Total | Prevalence and 95 CI | I2 (%) | Events | Total | Prevalence and 95 CI | I2 (%) | Events | Total | Prevalence and 95 CI | I2 (%) |  |
| Rash | 1870 | 1966 | 0.968 (0.905-1.000) | 94 | 7470 | 7983 | 0.953 (0.906-1.000) | 97.4 | 9 | 9 | 1.000 (0.844-1.000) | 0 | 1520 | 1680 | 0.954 (0.900-1.000) | 91.6 | 165 | 175 | 0.944 (0.908-0.980) | 0 | *.94* |
| Fever | 1824 | 1863 | 0.945 (0.899-0.990) | 80.3 | 2672 | 4956 | 0.683 (0.559-0.808) | 93.4 | 7 | 9 | 0.813 (0.571-1.000) | 0 | 938 | 1607 | 0.594 (0.538-0.651) | 71.2 | 139 | 175 | 0.800 (0.741-0.859) | 0 | *< .01* |
| Headache | 1216 | 1501 | 0.680 (0.517-0.843) | 98.4 | 2995 | 7639 | 0.492 (0.403-0.581) | 95.4 | 2 | 2 | 1.000 (0.158-1.000) | / | 429 | 1205 | 0.368 (0.297-0.439) | 81 | 41 | 172 | 0.237 (0.174-0.301) | 0 | *< .01* |
| Malaise/bedridden status/asthenia | 119 | 1392 | 0.441 (0.267-0.614) | 96.3 | 3391 | 7777 | 0.487 (0.338-0.636) | 95 | 2 | 2 | 1.000 (0.158-1.000) | / | 152 | 337 | 0.451 (0.284-0.617) | 82.4 | / | / | / | / | *.11* |
| Genital ulcers | 106 | 343 | 0.440 (0.212-0.668) | 91.7 | 2668 | 6160 | 0.398 (0.331-0.465) | 95.6 | 3 | 7 | 0.429 (0.099-0.816) | / | 407 | 902 | 0.496 (0.381-0.602) | 94.7 | / | / | / | / | *.55* |
| Lymphadenopathy | 1459 | 1940 | 0.700 (0.607-0.793) | 94.7 | 1131 | 2748 | 0.550 (0.386-0.715) | 97.9 | 7 | 9 | 0.823 (0.551-1.000) | 10.4 | 1013 | 1672 | 0.618 (0.551-0.685) | 90.1 | / | / | / | / | *.18* |
| Chill/rigors | 930 | 1149 | 0.739 (0.553-0.925) | 94.4 | 2844 | 6976 | 0.500 (0.379-0.621) | 96.2 | 1 | 2 | 0.500 (0.013-0.987) | / | / | / | / | / | 1 | 9 | 0.111 (0.003-0.482) | / | *< .01* |
| Enlarged lymph nodes | 227 | 282 | 0.805 (0.754-0.850) | / | 2040 | 5731 | 0.380 (0.316-0.443) | 81.7 | / | / | / | / | 326 | 558 | 0.613 (0.493-0.732) | 88.8 | / | / | / | / | *< .01* |
| Myalgia | 835 | 1146 | 0.549 (0.256-0.842) | 94.5 | 998 | 1993 | 0.497 (0.439-0.556) | 73.4 | 1 | 2 | 0.500 (0.013-0.987) | / | 324 | 872 | 0.435 (0.342-0.529) | 81.1 | 70 | 173 | 0.447 (0.132-0.762) | 64.2 | *.83* |
| Perianal ulcer | / | / | / | / | 1920 | 6159 | 0.359 (0.224-0.494) | 98.5 | / | / | / | / | 232 | 614 | 0.385 (0.316-0.453) | 57.2 | / | / | / | / | *.74* |
| Dysuria | / | / | / | / | 2069 | 5676 | 0.459 (0.249-0.668) | 98 | 1 | 2 | 0.500 (0.013-0.987) | / | / | / | / | / | / | / | / | / | *.91* |
| Fatigue | 974 | 1191 | 0.662 (0.488-0.836) | 95.6 | 106 | 206 | 0.661 (0.221-1.000) | 60.1 | 2 | 2 | 1.000 (0.158-1.000) | / | 24 | 56 | 0.429 (0.299-0.558) | 0 | 1 | 9 | 0.111 (0.003-0.482) | / | *< .01* |
| Body pain | 36 | 80 | 0.400 (0.160-0.639) | 83.2 | 1000 | 7523 | 0.280 (0.130-0.430) | 99 | / | / | / | / | 95 | 366 | 0.293 (0.000-0.607) | 97.8 | / | / | / | / | *.70* |
| Sore throat | 457 | 815 | 0.515 (0.432-0.599) | 72 | 275 | 961 | 0.426 (0.234-0.619) | 91.2 | / | / | / | / | 245 | 1146 | 0.249 (0.170-0.328) | 85.5 | 2 | 10 | 0.510 (0.000-1.000) | 86.5 | *< .01* |
| Nausea/vomiting | 302 | 1494 | 0.145 (0.073-0.218) | 94.9 | 659 | 6764 | 0.340 (0.085-0.596) | 91.6 | / | / | / | / | / | / | / | / | / | / | / | / | *.15* |
| Mouth ulcers | 796 | 1503 | 0.519 (0.383-0.655) | 88.8 | 179 | 1007 | 0.178 (0.155-0.203) | / | 1 | 7 | 0.143 (0.004-0.579) | / | 77 | 455 | 0.149 (0.046-0.252) | 89.2 | / | / | / | / | *< .01* |
| Itching | 718 | 1195 | 0.595 (0.449-0.742) | 84.7 | 319 | 925 | 0.426 (0.013-0.838) | 97.7 | 2 | 2 | 1.000 (0.158-1.000) | / | 2 | 3 | 0.740 (0.250-1.000) | 29.2 | / | / | / | / | *.23* |
| Perioral | / | / | / | / | 845 | 5069 | 0.332 (0.000-0.797) | 99.8 | / | / | / | / | 81 | 370 | 0.196 (0.092-0.300) | 87.5 | / | / | / | / | *.58* |
| Cough | 850 | 1818 | 0.369 (0.266-0.472) | 92.9 | 143 | 960 | 0.447 (0.082-0.812) | 95.2 | / | / | / | / | 8 | 122 | 0.060 (0.018-0.102) | 0 | / | / | / | / | *< .01* |
| Proctalgia/diarrhea | 54 | 626 | 0.083 (0.037-0.129) | 78.5 | 725 | 7394 | 0.124 (0.069-0.179) | 96.2 | / | / | / | / | 77 | 329 | 0.227 (0.090-0.365) | 89.5 | / | / | / | / | *.11* |
| Rectal bleeding | / | / | / | / | 910 | 7398 | 0.102 (0.053-0.150) | 96.1 | / | / | / | / | 12 | 20 | 0.600 (0.361-0.809) | / | / | / | / | / | *< .01* |
| Pus or blood in stools | / | / | / | / | 863 | 7347 | 0.117 (0.062-0.173) | 95.4 | / | / | / | / | / | / | / | / | / | / | / | / | */* |
| Tenesmus | 3 | 88 | 0.034 (0.007-0.096) | / | 801 | 7339 | 0.106 (0.084-0.129) | 75.2 | / | / | / | / | / | / | / | / | / | / | / | / | *< .01* |
| Dysphagia/swallowing | 755 | 1051 | 0.855 (0.574-1.000) | 98.3 | 4 | 34 | 0.118 (0.033-0.275) | / | / | / | / | / | 47 | 166 | 0.279 (0.211-0.347) | 0 | / | / | / | / | *< .01* |
| Conjunctivities | 311 | 1500 | 0.223 (0.169-0.276) | 66.9 | 167 | 3050 | 0.055 (0.047-0.063) | 0 | / | / | / | / | / | / | / | / | / | / | / | / | *< .01* |
| Corneal opacities/photophobia | 382 | 1787 | 0.162 (0.037-0.287) | 99.2 | 38 | 719 | 0.053 (0.038-0.072) | / | / | / | / | / | 32 | 334 | 0.187 (0.000-0.511) | 96.9 | / | / | / | / | *.17* |
| Sweating/joint pain | 94 | 147 | 0.552 (0.210-0.894) | 83.6 | 341 | 960 | 0.453 (0.300-0.606) | 67.1 | / | / | / | / | / | / | / | / | / | / | / | / | *.61* |
| Tonsillitis | 145 | 282 | 0.514 (0.454-0.574) | / | / | / | / | / | / | / | / | / | 36 | 301 | 0.117 (0.081-0.153) | 0 | / | / | / | / | *< .01* |
| Nasal congestion/rhinorrhea | 5 | 40 | 0.125 (0.042-0.268) | / | 134 | 958 | 0.148 (0.007-0.289) | 96.8 | / | / | / | / | / | / | / | / | / | / | / | / | *.80* |
| Arthralgia | / | / | / | / | / | / | / | / | / | / | / | / | 44 | 227 | 0.324 (0.000-0.750) | 96.6 | 36 | 163 | 0.221 (0.160-0.292) | / | *.64* |
| Flu-like symptoms | / | / | / | / | / | / | / | / | / | / | / | / | 88 | 179 | 0.492 (0.416-0.567) | / | / | / | / | / | */* |
| Swelling | 43 | 99 | 0.331 (0.000-0.694) | 93.4 | 2 | 34 | 0.059 (0.007-0.197) | / | / | / | / | / | / | / | / | / | / | / | / | / | *.15* |
| Difficulty breathing | 12 | 19 | 0.632 (0.384-0.837) | / | / | / | / | / | / | / | / | / | 31 | 264 | 0.117 (0.081-0.163) | / | / | / | / | / | *< .01* |
| Hepatomegaly | 31 | 303 | 0.102 (0.068-0.136) | 0 | / | / | / | / | / | / | / | / | / | / | / | / | / | / | / | / | */* |
| Adenopathy | / | / | / | / | 24 | 34 | 0.706 (0.525-0.749) | / | / | / | / | / | / | / | / | / | / | / | / | / | */* |
| Dehydration | 19 | 303 | 0.062 (0.035-0.089) | 0 | / | / | / | / | / | / | / | / | / | / | / | / | / | / | / | / | */* |
| Short of breath | 12 | 19 | 0.632 (0.384-0.837) | / | 6 | 34 | 0.176 (0.068-0.345) | / | / | / | / | / | / | / | / | / | / | / | / | / | *< .01* |
| Scrotal edema | 2 | 40 | 0.050 (0.006-0.169) | / | / | / | / | / | / | / | / | / | 10 | 22 | 0.455 (0.247-0.662) | 0 | / | / | / | / | *< .01* |
| Anorexia | 12 | 40 | 0.300 (0.000-0.722) | 90.7 | / | / | / | / | / | / | / | / | / | / | / | / | / | / | / | / | */* |
| Alopecia | 9 | 432 | 0.019 (0.006-0.032) | 0 | / | / | / | / | / | / | / | / | / | / | / | / | / | / | / | / | */* |
| Stiff neck | 1 | 6 | 0.167 (0.004-0.641) | / | 4 | 34 | 0.123 (0.022-0.225) | / | / | / | / | / | / | / | / | / | / | / | / | / | *.76* |
| Hemorrhagic skin lesions | 5 | 40 | 0.125 (0.042-0.268) | / | / | / | / | / | / | / | / | / | / | / | / | / | / | / | / | / | */* |
| Ear pain | / | / | / | / | 3 | 34 | 0.088 (0.019-0.237) | / | / | / | / | / | / | / | / | / | / | / | / | / | */* |
| Wheezing | / | / | / | / | 3 | 34 | 0.088 (0.019-0.237) | / | / | / | / | / | / | / | / | / | / | / | / | / | */* |
| Diarrhea | 2 | 19 | 0.105 (0.013-0.331) | / | 1 | 1 | 1.000 (0.025-1.000) | / | / | / | / | / | / | / | / | / | / | / | / | / | *< .01* |
| Confusion | / | / | / | / | 2 | 34 | 0.059 (0.007-0.197) | / | / | / | / | / | / | / | / | / | / | / | / | / | */* |
| Seizures | / | / | / | / | 1 | 34 | 0.029 (0.001-0.153) | / | / | / | / | / | / | / | / | / | / | / | / | / | */* |
| Tongue sores | 2 | 21 | 0.095 (0.012-0.304) | / | / | / | / |  | / | / | / | / | / | / | / | / | / | / | / | / | */* |

The use of the symbol "/" in the "Event," "Total," and "Prevalence and 95 CI" columns indicates that no data was reported. In the "I2" column, it means that heterogeneity cannot be calculated because there was only one included study. In the "P" column, it means that the difference cannot be calculated because there was only one subgroup.

**Table S6 Monkeypox cases and distribution in the world during 1970-2002**

a

| Years | Democratic Republic of the Congo (DRC) | | | Liberia | | | Sierra Leone | | | Nigeria | | | Ivory Coast | | |
| --- | --- | --- | --- | --- | --- | --- | --- | --- | --- | --- | --- | --- | --- | --- | --- |
|  | Report cases | Confirmed cases | Death cases | Report cases | Confirmed cases | Death cases | Report cases | Confirmed cases | Death cases | Report cases | Confirmed cases | Death cases | Report cases | Confirmed cases | Death cases |
| 1970 | 1 | 1 | 1 | 4 | 4 |  | 1 | 1 |  |  |  |  |  |  |  |
| 1971 |  |  |  |  |  |  |  |  |  | 2 | 2 |  | 1 | 1 |  |
| 1972 | 5 | 5 |  |  |  |  |  |  |  |  |  |  |  |  |  |
| 1973 | 3 | 3 |  |  |  |  |  |  |  |  |  |  |  |  |  |
| 1974 | 1 | 1 |  |  |  |  |  |  |  |  |  |  |  |  |  |
| 1975 | 3 | 3 |  |  |  |  |  |  |  |  |  |  |  |  |  |
| 1976 | 5 | 5 |  |  |  |  |  |  |  |  |  |  |  |  |  |
| 1977 | 6 | 6 |  |  |  |  |  |  |  |  |  |  |  |  |  |
| 1978 | 12 | 12 |  |  |  |  |  |  |  | 1 | 1 |  |  |  |  |
| 1979 | 7 | 7 |  |  |  |  |  |  |  |  |  |  |  |  |  |
| 1980 | 3 | 3 |  |  |  |  |  |  |  |  |  |  |  |  |  |
| 1981 | 6 | 6 | 9.8% |  |  |  |  |  |  |  |  |  | 1 | 1 |  |
| 1982 | 37 | 37 | 9.8% |  |  |  |  |  |  |  |  |  |  |  |  |
| 1983 | 56 | 56 | 9.8% |  |  |  |  |  |  |  |  |  |  |  |  |
| 1984 | 92 | 92 | 9.8% |  |  |  |  |  |  |  |  |  |  |  |  |
| 1985 | 62 | 62 | 9.8% |  |  |  |  |  |  |  |  |  |  |  |  |
| 1986 | 56 | 56 | 9.8% (total 33 from 1981-1986) |  |  |  |  |  |  |  |  |  |  |  |  |
| 1987 |  |  |  |  |  |  |  |  |  |  |  |  |  |  |  |
| 1988 |  |  |  |  |  |  |  |  |  |  |  |  |  |  |  |
| 1989 |  |  |  |  |  |  |  |  |  |  |  |  |  |  |  |
| 1990 |  |  |  |  |  |  |  |  |  |  |  |  |  |  |  |
| 1991 |  |  |  |  |  |  |  |  |  |  |  |  |  |  |  |
| 1992 | 13 |  |  |  |  |  |  |  |  |  |  |  |  |  |  |
| 1993 |  |  |  |  |  |  |  |  |  |  |  |  |  |  |  |
| 1994 |  |  |  |  |  |  |  |  |  |  |  |  |  |  |  |
| 1995 | 32 |  |  |  |  |  |  |  |  |  |  |  |  |  |  |
| 1996 | 92 | 11 | 1%-5% |  |  |  |  |  |  |  |  |  |  |  |  |
| 1997 | 419 |  | 1%-5% (total 8 from 1996-1997) |  |  |  |  |  |  |  |  |  |  |  |  |
| 1998 |  |  |  |  |  |  |  |  |  |  |  |  |  |  |  |
| 1999 |  |  | 315 (data in doubt) |  |  |  |  |  |  |  |  |  |  |  |  |
| 2000 |  |  |  |  |  |  |  |  |  |  |  |  |  |  |  |
| 2001 | 388 | 4 | 13 |  |  |  |  |  |  |  |  |  |  |  |  |
| 2002 | 881 | 2 | 14 |  |  |  |  |  |  |  |  |  |  |  |  |
| Total | 2180 | 372 | 384 | 4 | 4 | 0 | 1 | 1 | 0 | 3 | 3 | 0 | 2 | 2 | 0 |

b

| Years | Cameroon | | | Central African Republic (CAR) | | | Gabon | | | Total | | |
| --- | --- | --- | --- | --- | --- | --- | --- | --- | --- | --- | --- | --- |
|  | Report cases | Confirmed cases | Death cases | Report cases | Confirmed cases | Death cases | Report cases | Confirmed cases | Death cases | Report cases | Confirmed cases | Death cases |
| 1970 |  |  |  |  |  |  |  |  |  | 6 | 6 | 1 |
| 1971 |  |  |  |  |  |  |  |  |  | 3 | 3 | 0 |
| 1972 |  |  |  |  |  |  |  |  |  | 5 | 5 | 3 |
| 1973 |  |  |  |  |  |  |  |  |  | 3 | 3 | 1 |
| 1974 |  |  |  |  |  |  |  |  |  | 1 | 1 | 0 |
| 1975 |  |  |  |  |  |  |  |  |  | 3 | 3 | 0 |
| 1976 |  |  |  |  |  |  |  |  |  | 5 | 5 | 0 |
| 1977 |  |  |  |  |  |  |  |  |  | 6 | 6 | 0 |
| 1978 |  |  |  |  |  |  |  |  |  | 13 | 13 | 3 |
| 1979 | 2 | 2 |  |  |  |  |  |  |  | 10 | 9 | 1 |
| 1980 |  |  |  |  |  |  |  |  |  | 4 | 3 | 0 |
| 1981 |  |  |  |  |  |  |  |  |  | 8 | 7 | 1 |
| 1982 |  |  |  |  |  |  |  |  |  | 40 | 37 | 4 |
| 1983 |  |  |  |  |  |  |  |  |  | 84 | 56 | 6 |
| 1984 |  |  |  | 5 | 5 |  |  |  |  | 97 | 97 | 10 |
| 1985 |  |  |  |  |  |  |  |  |  | 62 | 62 | 6 |
| 1986 |  |  |  |  |  |  |  |  |  | 56 | 56 | 6 |
| 1987 |  |  |  |  |  |  | 1 | 1 | 1 | 1 |  | 1 |
| 1988 |  |  |  |  |  |  |  |  |  | 0 |  | 0 |
| 1989 | 1 | 1 |  |  |  |  |  |  |  | 1 |  | 0 |
| 1990 | 4 | 1 |  |  |  |  |  |  |  | 4 |  | 0 |
| 1991 |  |  |  |  |  |  | 9 |  | 0 | 9 |  | 0 |
| 1992 |  |  |  |  |  |  |  |  |  | 13 |  | 0 |
| 1993 |  |  |  |  |  |  |  |  |  | 0 |  | 0 |
| 1994 |  |  |  |  |  |  |  |  |  | 0 |  | 0 |
| 1995 |  |  |  |  |  |  |  |  |  | 32 |  | 0 |
| 1996 |  |  |  |  |  |  |  |  |  | 92 | 11 | 3 |
| 1997 |  |  |  |  |  |  |  |  |  | 419 | 10 | 2 |
| 1998 |  |  |  |  |  |  |  |  |  | 0 |  | 0 |
| 1999 |  |  |  |  |  |  |  |  |  | 0 |  | 315 |
| 2000 |  |  |  |  |  |  |  |  |  | 0 |  | 0 |
| 2001 |  |  |  | 8 |  | 2 |  |  |  | 396 | 4 | 15 |
| 2002 |  |  |  |  |  |  |  |  |  | 881 | 2 | 37 |
| Total | 7 | 4 | 0 | 13 | 5 | 2 | 10 | 1 | 1 | 2254 | 399 | 415 |

*Monkeypox cases and distribution in the world during 1970-2002*

The first monkeypox (MPX) case was a 9-month-old child in DRC in August 1970. Subsequently, 4 and 1 cases were reported in Liberia and Sierra Leone, respectively. In April, 1972 cases were confirmed in Nigeria, October of the same year, 1 case was in Ivory Coast [62]. In the next six years, a total of 23 cases (4 deaths) were reported in DRC [62]. Later, 23 cases were reported in 1978-1979 by WHO [63], including 19 cases (4 deaths) in DRC, 2 cases in Cameroon, 1 case in Nigeria [64], and 1 case unknown. Consequently, 3 cases were reported in DRC in 1980. Between 1981 and 1986, a total of 347 cases were confirmed by MPX[63], and the case fatality rate (CFR) was 9.8% (33 deaths) [65]. From 1987-1992, MPX cases significantly decreased due to discontinued surveillance. A total of 28 cases (1 death) were reported in Gabon (10), Carmeroon (4), and DRC (13).[66, 67] No cases were reported between 1993-1994. Until 1995, 32 cases were reported in DRC [66], and a total of 511 cases were occurred between February 1996 and October 1997, the CFR was about 1%-5% (5 deaths) [68]. From 1998-2000, no clear data were reported, only 315 deaths in DRC were reported in 1999 [69], while the number increased to 388 cases (13 deaths) in 2001 and 881 (14 deaths) in 2002 in DRC.

**Table S7 Monkeypox cases and distribution in the world during 2003-2021.**

a

| Years | Democratic Republic of the Congo (DRC) | | | Liberia | | | Sierra Leone | | | Nigeria | | | Ivory Coast | | |
| --- | --- | --- | --- | --- | --- | --- | --- | --- | --- | --- | --- | --- | --- | --- | --- |
|  | Report cases | Confirmed cases | Death cases | Report cases | Confirmed cases | Death cases | Report cases | Confirmed cases | Death cases | Report cases | Confirmed cases | Death cases | Report cases | Confirmed cases | Death cases |
| 2003 | 755 |  | 16 |  |  |  |  |  |  |  |  |  |  |  |  |
| 2004 | 1024 |  | 29 |  |  |  |  |  |  |  |  |  |  |  |  |
| 2005 | 1708 |  | 26 |  |  |  |  |  |  |  |  |  |  |  |  |
| 2006 | 783 |  | 20 |  |  |  |  |  |  |  |  |  |  |  |  |
| 2007 | 970 | 760 | 11 |  |  |  |  |  |  |  |  |  |  |  |  |
| 2008 | 1599 |  | 67 |  |  |  |  |  |  |  |  |  |  |  |  |
| 2009 | 1919 |  | 27 |  |  |  |  |  |  |  |  |  |  |  |  |
| 2010 | 2322 |  | 26 |  |  |  |  |  |  |  |  |  |  |  |  |
| 2011 | 2208 | 3 | 15 |  |  |  |  |  |  |  |  |  |  |  |  |
| 2012 | 2629 |  | 34 |  |  |  |  |  |  |  |  |  |  |  |  |
| 2013 | 2460 |  | 37 |  |  |  |  |  |  |  |  |  |  |  |  |
| 2014 | 423 |  |  |  |  |  | 1 |  |  |  |  |  |  |  |  |
| 2015 | 587 |  |  |  |  |  |  |  |  |  |  |  |  |  |  |
| 2016 | 195 | 1 | 11 |  |  |  |  |  |  |  |  |  |  |  |  |
| 2017 |  |  |  | 16 |  | 2 | 1 |  |  | 144 | 88 |  |  |  |  |
| 2018 | 2995 | 34 | 36 |  |  |  |  |  |  | 116 | 49 |  |  |  |  |
| 2019 | 3794 |  |  |  |  |  |  |  |  | 65 | 47 |  |  |  |  |
| 2020 | 4594 |  |  |  |  |  |  |  |  | 35 | 8 |  |  |  |  |
| 2021 | 2708 |  |  |  |  |  |  |  |  | 25 | 3 |  |  |  |  |
| Total | 33673 | 798 | 355 | 16 | 0 | 2 | 2 | 0 | 0 | 385 | 195 | 0 | 0 | 0 | 0 |

b

| Years | Cameroon | | | Central African Republic (CAR) | | | Gabon | | | Republic of the Congo (ROC) | | | South Sudan | | |
| --- | --- | --- | --- | --- | --- | --- | --- | --- | --- | --- | --- | --- | --- | --- | --- |
|  | Report cases | Confirmed cases | Death cases | Report cases | Confirmed cases | Death cases | Report cases | Confirmed cases | Death cases | Report cases | Confirmed cases | Death cases | Report cases | Confirmed cases | Death cases |
| 2003 |  |  |  |  |  |  |  |  |  | 12 | 3 | 1 |  |  |  |
| 2004 |  |  |  |  |  |  |  |  |  |  |  |  |  |  |  |
| 2005 |  |  |  |  |  |  |  |  |  |  |  |  | 49 | 10 |  |
| 2006 |  |  |  |  |  |  |  |  |  |  |  |  |  |  |  |
| 2007 |  |  |  |  |  |  |  |  |  | 62 | 62 |  |  |  |  |
| 2008 |  |  |  |  |  |  |  |  |  |  |  |  |  |  |  |
| 2009 |  |  |  |  |  |  |  |  |  |  |  |  |  |  |  |
| 2010 |  |  |  | 2 | 2 |  |  |  |  | 12 | 2 | 1 |  |  |  |
| 2011 |  |  |  |  |  |  |  |  |  |  |  |  |  |  |  |
| 2012 |  |  |  |  |  |  |  |  |  |  |  |  |  |  |  |
| 2013 |  |  |  |  |  |  |  |  |  |  |  |  |  |  |  |
| 2014 |  |  |  |  |  |  |  |  |  |  |  |  |  |  |  |
| 2015 |  |  |  | 13 | 13 | 3 |  |  |  |  |  |  |  |  |  |
| 2016 |  |  |  | 52 | 3 | 3 |  |  |  |  |  |  |  |  |  |
| 2017 |  |  |  | 17 | 8 | 1 |  |  |  | 88 | 7 | 6 |  |  |  |
| 2018 | 16 | 1 | 1 | 14 | 9 | 0 |  |  |  |  |  |  |  |  |  |
| 2019 |  |  |  |  |  |  |  |  |  |  |  |  |  |  |  |
| 2020 |  |  |  |  |  |  |  |  |  |  |  |  |  |  |  |
| 2021 |  |  |  |  |  |  |  |  |  |  |  |  |  |  |  |
| Total | 16 | 1 | 1 | 98 | 35 | 7 | 0 | 0 | 0 | 174 | 74 | 8 | 49 | 10 | 0 |

c

| Years | Israel | | | United States (US) | | | United Kingdom (UK) | | | Singapore | | | Total | | |
| --- | --- | --- | --- | --- | --- | --- | --- | --- | --- | --- | --- | --- | --- | --- | --- |
|  | Report cases | Confirmed cases | Death cases | Report cases | Confirmed cases | Death cases | Report cases | Confirmed cases | Death cases | Report cases | Confirmed cases | Death cases | Report cases | Confirmed cases | Death cases |
| 2003 |  |  |  | 81 | 41 |  |  |  |  |  |  |  | 848 | 40 | 17 |
| 2004 |  |  |  |  |  |  |  |  |  |  |  |  | 1024 |  | 29 |
| 2005 |  |  |  |  |  |  |  |  |  |  |  |  | 1757 |  | 26 |
| 2006 |  |  |  |  |  |  |  |  |  |  |  |  | 783 |  | 20 |
| 2007 |  |  |  |  |  |  |  |  |  |  |  |  | 1032 | 822 | 11 |
| 2008 |  |  |  |  |  |  |  |  |  |  |  |  | 1599 |  | 67 |
| 2009 |  |  |  |  |  |  |  |  |  |  |  |  | 1919 |  | 27 |
| 2010 |  |  |  |  |  |  |  |  |  |  |  |  | 2336 | 4 | 27 |
| 2011 |  |  |  |  |  |  |  |  |  |  |  |  | 2208 | 3 | 15 |
| 2012 |  |  |  |  |  |  |  |  |  |  |  |  | 2629 |  | 34 |
| 2013 |  |  |  |  |  |  |  |  |  |  |  |  | 2460 |  | 37 |
| 2014 |  |  |  |  |  |  |  |  |  |  |  |  | 424 | 1 | 2 |
| 2015 |  |  |  |  |  |  |  |  |  |  |  |  | 600 | 12 | 3 |
| 2016 |  |  |  |  |  |  |  |  |  |  |  |  | 247 | 4 | 14 |
| 2017 |  |  |  |  |  |  |  |  |  |  |  |  | 266 | 86 | 9 |
| 2018 | 1 | 1 |  |  |  |  | 2 | 2 |  |  |  |  | 3144 | 147 | 60 |
| 2019 |  |  |  |  |  |  |  |  |  | 1 | 1 |  | 3860 | 47 | 73 |
| 2020 |  |  |  |  |  |  |  |  |  |  |  |  | 4629 |  | 171 |
| 2021 |  |  |  | 1 | 1 |  | 3 | 3 |  |  |  |  | 2737 | 4 | 75 |
| Total | 1 | 1 | 0 | 82 | 42 | 0 | 5 | 5 | 0 | 1 | 1 | 0 | 34502 | 1170 | 717 |

*Monkeypox cases and distribution in the world during 2003-2021*

The United States has first reported 82 MPX case in 2003. Regarding DRC, the outbreak continued which has led to 755 (16 deaths) in 2003, 1024 (29 deaths) in 2004, 1708 (26 deaths) in 2005, 783 (20 deaths) in 2006, 970 (760 confirmed, 11 deaths) in 2007, 1599 (67 deaths) in 2008, 1919 (27 deaths) in 2009, 2322 (26 deaths) in 2010, 2208 (15 deaths) in 2011, 2629 (34 deaths) in 2012, and 2460 (37 deaths) in 2013 [70]. In 2001 and 2010, 8 cases (2 deaths) and 2 confirmed cases were reported respectively in CAR [71]. In 2003, 2007 and 2010, 12 cases (3 confirmed, 1 death) [72], 62 confirmed cases [73], 11 cases (2 confirmed, 1 death) [74] were reported respectively in ROC. In 2005, 49 cases (10 confirmed) were described in South Sudan [6]. In addition, MPX emerged in USA which was the first outbreak country outside African. A total of 81 cases (41 confirmed) occurred in Illinois, Indiana, Kansas, Missouri, and Wisconsin state [66].

From 2014-2016, a total of 1205 cases reported in DRC, 423 cases in 2014, 567 in 2015, and 195 (11 deaths) in 2016 [75] [76]. 13 confirmed cases (3 deaths), 52 cases (3 confirmed, 3 deaths), 17 cases (3 confirmed, 3 deaths), and 14 cases (9 confirmed) were respectively reported in CAR from 2015 to 2018 [67]. 2 cases were reported in Sierra Leone from 2014-2017 [76] [67]. In 2017, 16 cases (2 deaths) were reported in Liberia [77], 88 cases (7 confirmed, 6 deaths) in ROC. In 2018, 16 cases (1 confirmed, 1 death) were reported in Liberia [78]. From 2017-2021, 144 cases (88 confirmed), 116 cases (49 confirmed), 65 cases (47 confirmed), 35 cases (8 confirmed) and 25 cases (3 confirmed) were sequentially described in Nigeria [79]. Between 2018 and 2021, 14091 cases were reported in DRC, 2995 cases in 2018, 3794 in 2019, 4594 in 2020, 2708 in 2021[80]. The cases in 2018-2021 were all imported cases. 1 case was reported in Israel in 2018[81], 1 case in Singapore in 2019[82], 1 case in USA in 2021 [83], 2 and 3 cases in the United Kingdom (UK) in 2018 and 2021[12].

**Table S8 Monkeypox prevalence in the world since 2022**

| Europe | Prevalence | Americas | Prevalence | Eastern Mediterranean | Prevalence | Western Pacific | Prevalence | Africa | Prevalence | South-East Asia | Prevalence |
| --- | --- | --- | --- | --- | --- | --- | --- | --- | --- | --- | --- |
| Gibraltar | 17.809 | Peru | 11.391 | Malta | 6.578 | New Zealand | 0.800 | Ghana | 0.391 | Thailand | 0.027 |
| Spain | 15.951 | United States | 9.082 | Israel | 2.798 | Guam | 0.588 | Sudan | 0.042 | India | 0.002 |
| Portugal | 9.253 | Colombia | 7.978 | Cyprus | 0.411 | Australia | 0.559 | Benin | 0.024 | Indonesia | 0.000 |
| Luxembourg | 8.919 | Chile | 7.495 | Lebanon | 0.399 | Singapore | 0.422 | Somalia | 0.018 |  |  |
| Monaco | 7.591 | Puerto Rico | 6.465 | Qatar | 0.171 | New Caledonia | 0.367 | South Sudan | 0.018 |  |  |
| Netherlands | 7.198 | Brazil | 5.094 | United Arab Emirates | 0.160 | Japan | 0.088 | Uganda | 0.013 |  |  |
| Belgium | 6.843 | Panama | 5.089 | Bahrain | 0.057 | South Korea | 0.025 | South Africa | 0.008 |  |  |
| Switzerland | 6.346 | Costa Rica | 4.281 | Georgia | 0.054 | Hong Kong, China | 0.013 | Morocco | 0.008 |  |  |
| France | 6.139 | Canada | 3.870 | Saudi Arabia | 0.023 | Taiwan, China | 0.013 | Zambia | 0.005 |  |  |
| United Kingdom | 5.556 | Greenland | 3.530 | Jordan | 0.010 | Philippines | 0.004 |  |  |  |  |
| Andorra | 5.171 | Mexico | 3.037 | Egypt | 0.003 | China | 0.002 |  |  |  |  |
| Ireland | 4.534 | Ecuador | 2.980 | Iran | 0.001 |  |  |  |  |  |  |
| Germany | 4.441 | Aruba | 2.799 | Pakistan | 0.000 |  |  |  |  |  |  |
| Iceland | 4.298 | Argentina | 2.462 |  |  |  |  |  |  |  |  |
| Austria | 3.662 | Guatemala | 2.361 |  |  |  |  |  |  |  |  |
| Denmark | 3.347 | Bolivia | 2.240 |  |  |  |  |  |  |  |  |
| Saint Martin (French part) | 2.548 | Curaçao | 1.969 |  |  |  |  |  |  |  |  |
| Sweden | 2.496 | Paraguay | 1.731 |  |  |  |  |  |  |  |  |
| Slovenia | 2.231 | El Salvador | 1.595 |  |  |  |  |  |  |  |  |
| Norway | 1.757 | Bermuda | 1.566 |  |  |  |  |  |  |  |  |
| Italy | 1.620 | Cayman Islands | 1.504 |  |  |  |  |  |  |  |  |
| Croatia | 0.846 | Jamaica | 0.706 |  |  |  |  |  |  |  |  |
| Estonia | 0.828 | Uruguay | 0.545 |  |  |  |  |  |  |  |  |
| Hungary | 0.824 | Bahamas | 0.504 |  |  |  |  |  |  |  |  |
| Greece | 0.816 | Dominican Republic | 0.475 |  |  |  |  |  |  |  |  |
| Finland | 0.758 | Honduras | 0.417 |  |  |  |  |  |  |  |  |
| Czech Republic | 0.607 | Barbados | 0.348 |  |  |  |  |  |  |  |  |
| Serbia | 0.584 | Guyana | 0.253 |  |  |  |  |  |  |  |  |
| Poland | 0.574 | Cuba | 0.071 |  |  |  |  |  |  |  |  |
| Montenegro | 0.322 | Venezuela | 0.042 |  |  |  |  |  |  |  |  |
| Latvia | 0.319 |  |  |  |  |  |  |  |  |  |  |
| Bosnia And Herzegovina | 0.276 |  |  |  |  |  |  |  |  |  |  |
| Slovakia | 0.257 |  |  |  |  |  |  |  |  |  |  |
| Romania | 0.246 |  |  |  |  |  |  |  |  |  |  |
| Lithuania | 0.179 |  |  |  |  |  |  |  |  |  |  |
| Bulgaria | 0.087 |  |  |  |  |  |  |  |  |  |  |
| Moldova | 0.078 |  |  |  |  |  |  |  |  |  |  |
| Turkey | 0.014 |  |  |  |  |  |  |  |  |  |  |
| Ukraine | 0.011 |  |  |  |  |  |  |  |  |  |  |
| Russia | 0.001 |  |  |  |  |  |  |  |  |  |  |

*Monkeypox cases and distribution in the world since 2022*

Since 2022, the largest MPX outbreak was confirmed in the USA (30,142 confirmed cases, 44 deaths), followed by Brazil (10,900 confirmed cases, 15 deaths), Spain (7,549 confirmed cases, 3 deaths), France (4,114 confirmed cases), Colombia (4,090 confirmed cases), Mexico (3,956 confirmed cases, 5 deaths), Peru (3,800 confirmed cases, 20 deaths), United Kingdom (3,741 confirmed cases), Germany (3,692 confirmed cases), Canada (1,480 confirmed cases), Chile (1,440 confirmed cases, 2 deaths), Netherlands (1,262 confirmed cases), Argentina (1,028 confirmed cases, 2 deaths). A total of 21 countries have confirmed cases between 100 and 1,000, such as Italy (957 confirmed cases), Portugal (953 confirmed cases), Belgium (793 confirmed cases, 2 deaths), and so on. 29 countries have confirmed cases between 10 and 100, such as Norway (95 confirmed cases), Greece (87 confirmed cases), Hungary (80 confirmed cases), and so on. Other 43 countries or areas have confirmed cases between 1 and 10, such as Bosnia and Herzegovina (9 confirmed cases), Cuba (8 confirmed cases, 1 death), Saudi Arabia (8 confirmed cases), etc.[84]

**Table S9 PRISMA Checklist**

| **Section and Topic** | **Item #** | **Checklist item** | **Location where item is reported** |
| --- | --- | --- | --- |
| **TITLE** | | |  |
| Title | 1 | Identify the report as a systematic review. | 1 |
| **ABSTRACT** | | |  |
| Abstract | 2 | See the PRISMA 2020 for Abstracts checklist. | 2 |
| **INTRODUCTION** | | |  |
| Rationale | 3 | Describe the rationale for the review in the context of existing knowledge. | 3 |
| Objectives | 4 | Provide an explicit statement of the objective(s) or question(s) the review addresses. | 4 |
| **METHODS** | | |  |
| Eligibility criteria | 5 | Specify the inclusion and exclusion criteria for the review and how studies were grouped for the syntheses. | 4 |
| Information sources | 6 | Specify all databases, registers, websites, organisations, reference lists and other sources searched or consulted to identify studies. Specify the date when each source was last searched or consulted. | 4 |
| Search strategy | 7 | Present the full search strategies for all databases, registers and websites, including any filters and limits used. | 4 |
| Selection process | 8 | Specify the methods used to decide whether a study met the inclusion criteria of the review, including how many reviewers screened each record and each report retrieved, whether they worked independently, and if applicable, details of automation tools used in the process. | 4 |
| Data collection process | 9 | Specify the methods used to collect data from reports, including how many reviewers collected data from each report, whether they worked independently, any processes for obtaining or confirming data from study investigators, and if applicable, details of automation tools used in the process. | 4 |
| Data items | 10a | List and define all outcomes for which data were sought. Specify whether all results that were compatible with each outcome domain in each study were sought (e.g. for all measures, time points, analyses), and if not, the methods used to decide which results to collect. | 4 |
|  | 10b | List and define all other variables for which data were sought (e.g. participant and intervention characteristics, funding sources). Describe any assumptions made about any missing or unclear information. | NA |
| Study risk of bias assessment | 11 | Specify the methods used to assess risk of bias in the included studies, including details of the tool(s) used, how many reviewers assessed each study and whether they worked independently, and if applicable, details of automation tools used in the process. | 5 |
| Effect measures | 12 | Specify for each outcome the effect measure(s) (e.g. risk ratio, mean difference) used in the synthesis or presentation of results. | 5 |
| Synthesis methods | 13a | Describe the processes used to decide which studies were eligible for each synthesis (e.g. tabulating the study intervention characteristics and comparing against the planned groups for each synthesis (item #5)). | 5 |
|  | 13b | Describe any methods required to prepare the data for presentation or synthesis, such as handling of missing summary statistics, or data conversions. | NA |
|  | 13c | Describe any methods used to tabulate or visually display results of individual studies and syntheses. | NA |
|  | 13d | Describe any methods used to synthesize results and provide a rationale for the choice(s). If meta-analysis was performed, describe the model(s), method(s) to identify the presence and extent of statistical heterogeneity, and software package(s) used. | 5 |
|  | 13e | Describe any methods used to explore possible causes of heterogeneity among study results (e.g. subgroup analysis, meta-regression). | 5 |
|  | 13f | Describe any sensitivity analyses conducted to assess robustness of the synthesized results. | 5-6 |
| Reporting bias assessment | 14 | Describe any methods used to assess risk of bias due to missing results in a synthesis (arising from reporting biases). | 5 |
| Certainty assessment | 15 | Describe any methods used to assess certainty (or confidence) in the body of evidence for an outcome. | 5 |
| **RESULTS** | | |  |
| Study selection | 16a | Describe the results of the search and selection process, from the number of records identified in the search to the number of studies included in the review, ideally using a flow diagram. | 6 |
|  | 16b | Cite studies that might appear to meet the inclusion criteria, but which were excluded, and explain why they were excluded. | 6 |
| Study characteristics | 17 | Cite each included study and present its characteristics. | 6-7 |
| Risk of bias in studies | 18 | Present assessments of risk of bias for each included study. | 6 |
| Results of individual studies | 19 | For all outcomes, present, for each study: (a) summary statistics for each group (where appropriate) and (b) an effect estimate and its precision (e.g. confidence/credible interval), ideally using structured tables or plots. | 7-9 |
| Results of syntheses | 20a | For each synthesis, briefly summarise the characteristics and risk of bias among contributing studies. | NA |
|  | 20b | Present results of all statistical syntheses conducted. If meta-analysis was done, present for each the summary estimate and its precision (e.g. confidence/credible interval) and measures of statistical heterogeneity. If comparing groups, describe the direction of the effect. | 7-9 |
|  | 20c | Present results of all investigations of possible causes of heterogeneity among study results. | NA |
|  | 20d | Present results of all sensitivity analyses conducted to assess the robustness of the synthesized results. | 9-10 |
| Reporting biases | 21 | Present assessments of risk of bias due to missing results (arising from reporting biases) for each synthesis assessed. | NA |
| Certainty of evidence | 22 | Present assessments of certainty (or confidence) in the body of evidence for each outcome assessed. | 6-10 |
| **DISCUSSION** | | |  |
| Discussion | 23a | Provide a general interpretation of the results in the context of other evidence. | 12-15 |
|  | 23b | Discuss any limitations of the evidence included in the review. | 15 |
|  | 23c | Discuss any limitations of the review processes used. | 15 |
|  | 23d | Discuss implications of the results for practice, policy, and future research. | 15 |
| **OTHER INFORMATION** | | |  |
| Registration and protocol | 24a | Provide registration information for the review, including register name and registration number, or state that the review was not registered. | 4 |
|  | 24b | Indicate where the review protocol can be accessed, or state that a protocol was not prepared. | 4 |
|  | 24c | Describe and explain any amendments to information provided at registration or in the protocol. | NA |
| Support | 25 | Describe sources of financial or non-financial support for the review, and the role of the funders or sponsors in the review. | 16 |
| Competing interests | 26 | Declare any competing interests of review authors. | 16 |
| Availability of data, code and other materials | 27 | Report which of the following are publicly available and where they can be found: template data collection forms; data extracted from included studies; data used for all analyses; analytic code; any other materials used in the review. | NA |

NA, not applicable.

**Figure S1 monkeypox prevalence in (a) 1970-2002, (b) 2003-2021, and (c) 2022-2023**


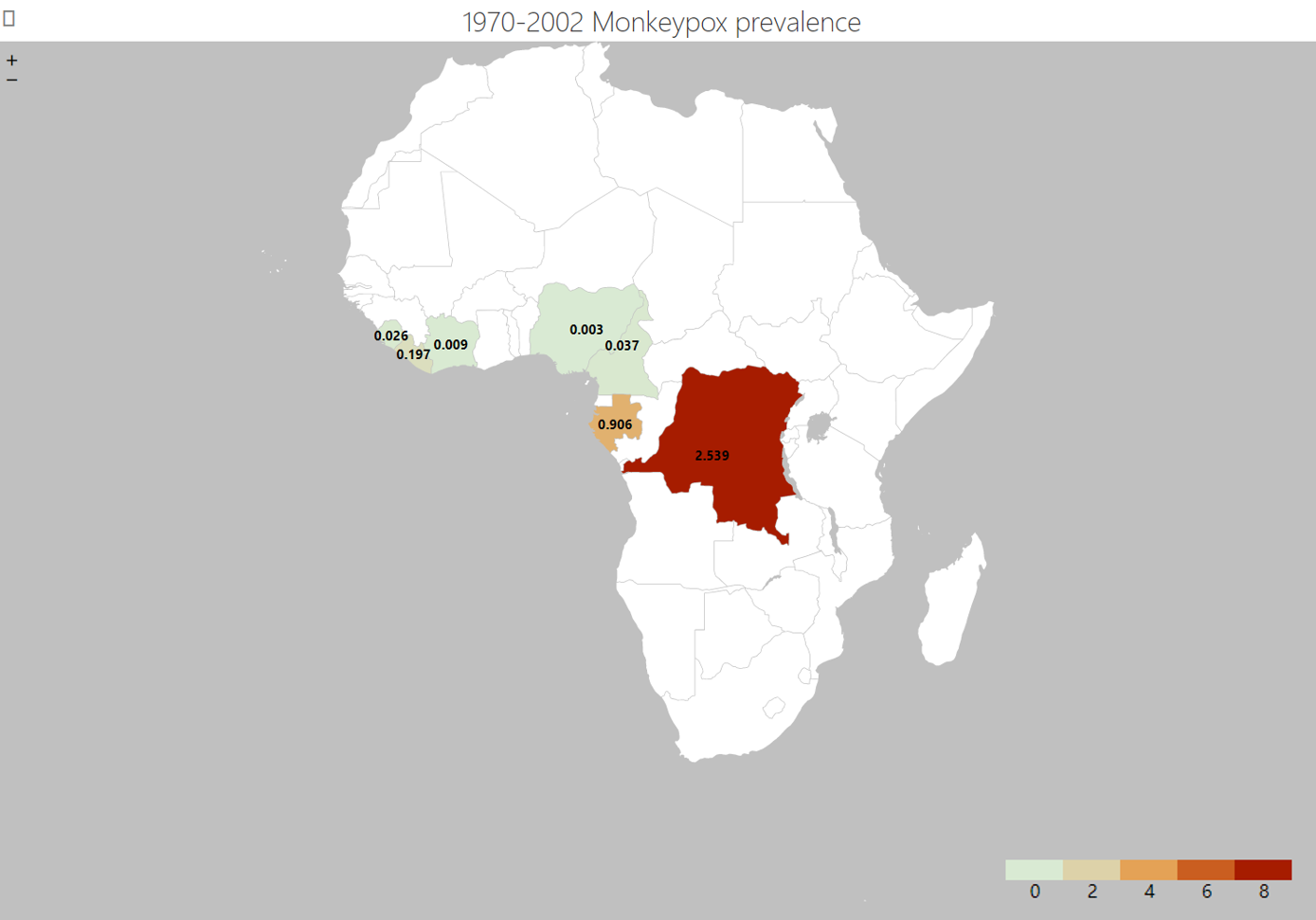


(a) Monkeypox prevalence from 1970-2002


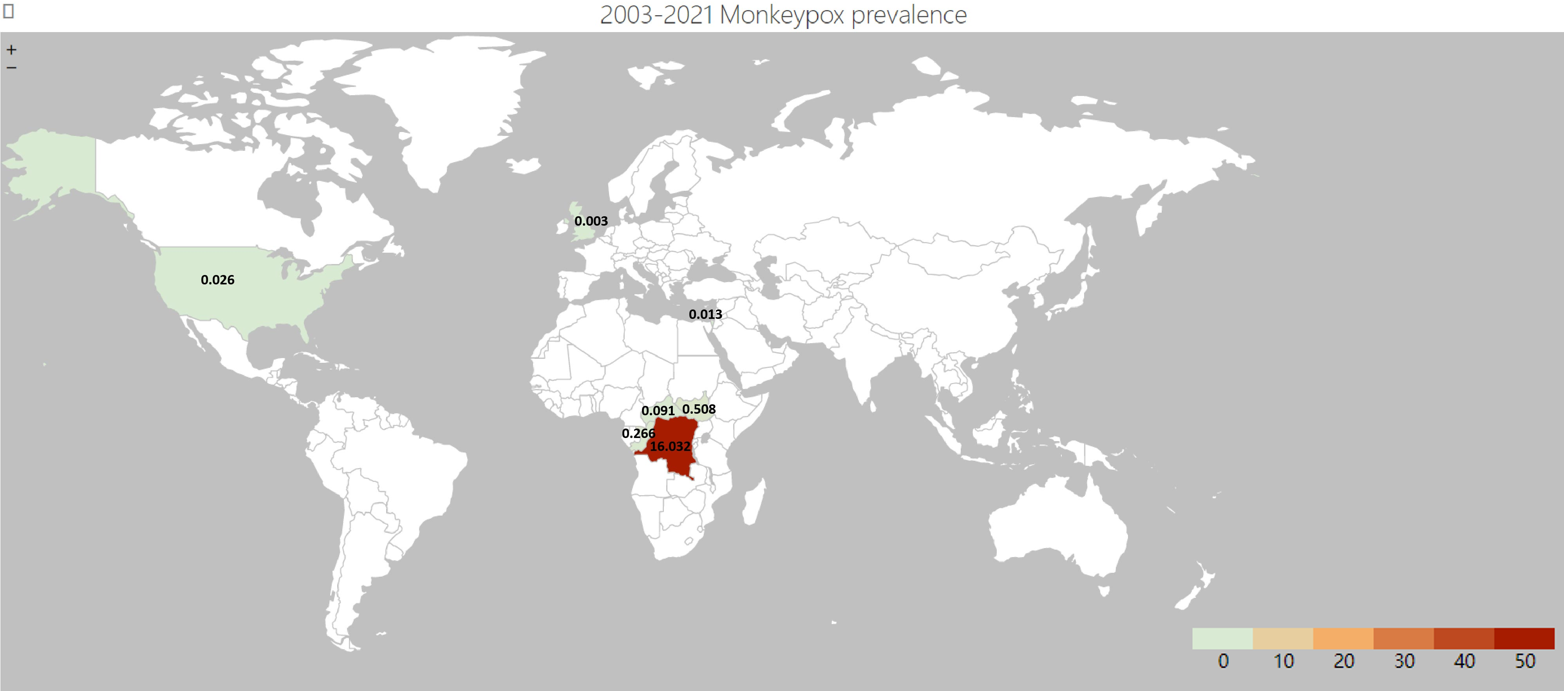


(b) Monkeypox prevalence from 2003-2021


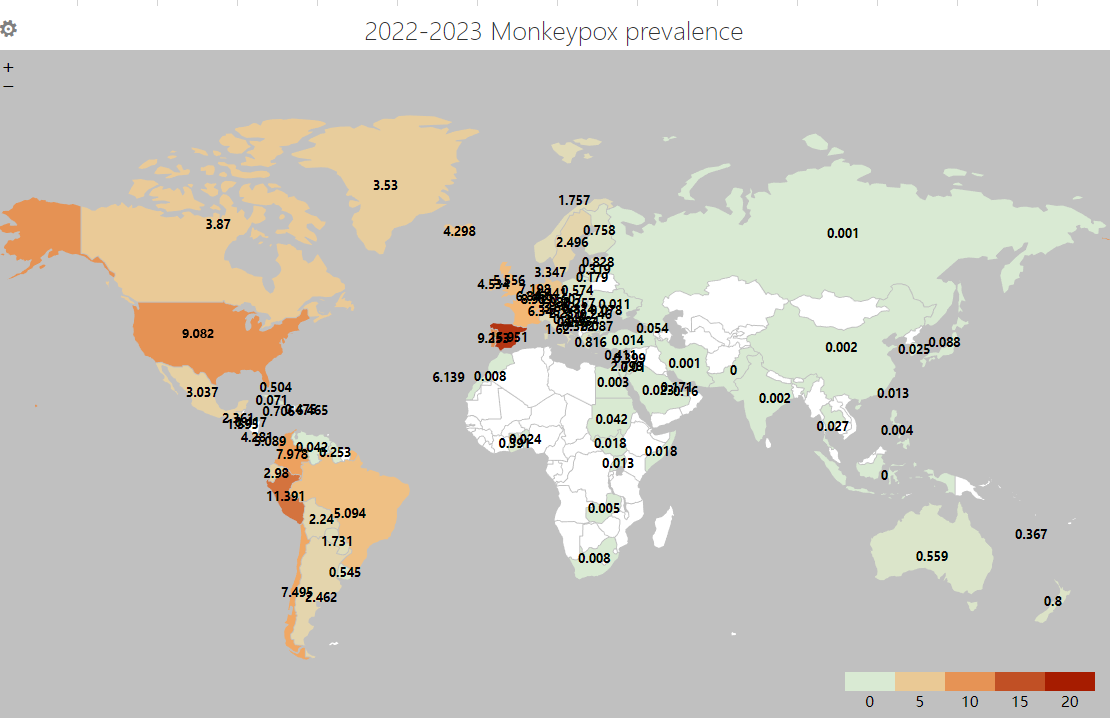


(c) Monkeypox prevalence since 2022

*Monkeypox prevalence in 1970-2002, 2003-2021, and 2022-2023*

The period prevalence was calculated to compare the disease burden between countries.

$$\frac{All new and pre-existing cases during a given period}{The population during the same period}\times{10}^{n}$$

The numerator is the number of current cases (new and preexisting) over a specified period. The denominator is the average population during the period, which was calculated based on World Bank population data.[85] The clinical symptom information of the patients and fatality rate were collected from the available data.

Since the first case was found in DRC [86], a total of 2254 cases had successively appeared in other 7 countries of central and western African during 1970 and 2002 (Appendix Table S3).[62-69] In this stage, the mean age of patients was 8.9±5.4 years old and the proportion of male was 51.1%。DRC had the highest prevalence (6.693/100000inhabitants), followed by Gabon (1.132), Central African Republic (CAR) (0.491), Liberia (0.197). Nigeria had the lowest prevalence (0.003). No cases were reported in other African countries. The MPX prevalence between 1970 and 2002 is shown in Appendix Figure S1a.

Since 2003, MPX first emerged outside Africa in United States. During this period, the mean age of patients were 16.4±9.2 and the proportion of male was 61.7%. Between 2003-2021, a total of 34502 cases were reported worldwide (Appendix Table S4), DRC still had the highest prevalence (48.037/100000inhabitants), followed by Republic of the Congo (ROC) (3.862) which was the newly outbreak country. The prevalence of CAR (2.223), Liberia (0.390), and Nigeria (0.228) were increased compared with 1970-2002 period. South Sudan (0.508) was a new outbreak country. Although the prevalence of Cameroon and Sierra Leone had increased compared with the period of 1970-2002, but they were still the two lowest African countries (0.074 and 0.030, respectively). Only 89 cases (46 cases confirmed) occurred in 4 countries outside African. United States (US) had the highest prevalence (0.026), followed by Singapore (0.020), Israel (0.013), and United Kingdom (UK) (0.008). The MPX prevalence between 2003 and 2021 is shown in Appendix Figure S1b.

Since 2022, MPX pandemic has been spreading all over the world. From January 1, 2022 to April 30, 2023, 105 WHO members in 6 regions countries have reported 87045 cases of MPX (Appendix Table S5). In this stage, the mean age of patients was 41.1 ±6.5 and the proportion of male was 98.6%. The highest prevalence countries were more distributed in Europe and Americas. A total of 40 Europe countries have emerged MPX cases, the highest prevalence countries were Gibraltar (17.809/100000inhabitants), Spain (15.951), Portugal (9.253), Luxembourg (8.919), Monaco (7.591), and Netherlands (7.198), and they were also the countries with the highest prevalence in the world, followed by Belgium (6.843), Switzerland (6.346), France (6.139), United Kingdom (5.556), Andorra (5.171), Ireland (4.534), Germany (4.312), and Iceland (4.298) and so on. Whereas the lowest prevalence countries in Europe were Bosnia and Herzegovina (0.091), Bulgaria (0.086), Moldova (0.077), Turkey (0.012), Ukraine (0.004), Russia (0.001), respectively. In 30 countries in Americas, Peru (11.391), United States (9.082), Colombia (7.978), and Chile (7.495) were the highest prevalence countries, followed by Puerto Rico (6.465), Brazil (5.094), Panama (5.089), and Costa Rica (4.281). It is worth mentioning that the prevalence in African countries were general lower compared with the previous period, the highest prevalence country was Ghana (0.391), followed by Sudan (0.042), Benin (0.024), Somalia (0.018), South Sudan (0.018), and Uganda (0.013). South Africa, Morocco, and Zambia were the lowest prevalence countries (0.008, 0.008 and 0.005, respectively). For Western Pacific, the highest prevalence was reported in New Zealand (0.800), Guam (0.588), followed by Australia (0.559), Singapore (0.422), and New Caledonia (0.367). The prevalence of Japan, South Korea, Philippines, and China were relatively low (0.088, 0.003, 0.025, 0.004, and 0.002, respectively). The prevalence of South-East Asia countries were Thailand (0.027) and India (0.002), lower than that of other regional countries. The MPX prevalence in 2022 is shown in Appendix Figure S1c.

**Figure S2 Sensitivity analysis result after excluding non-low risk studies**


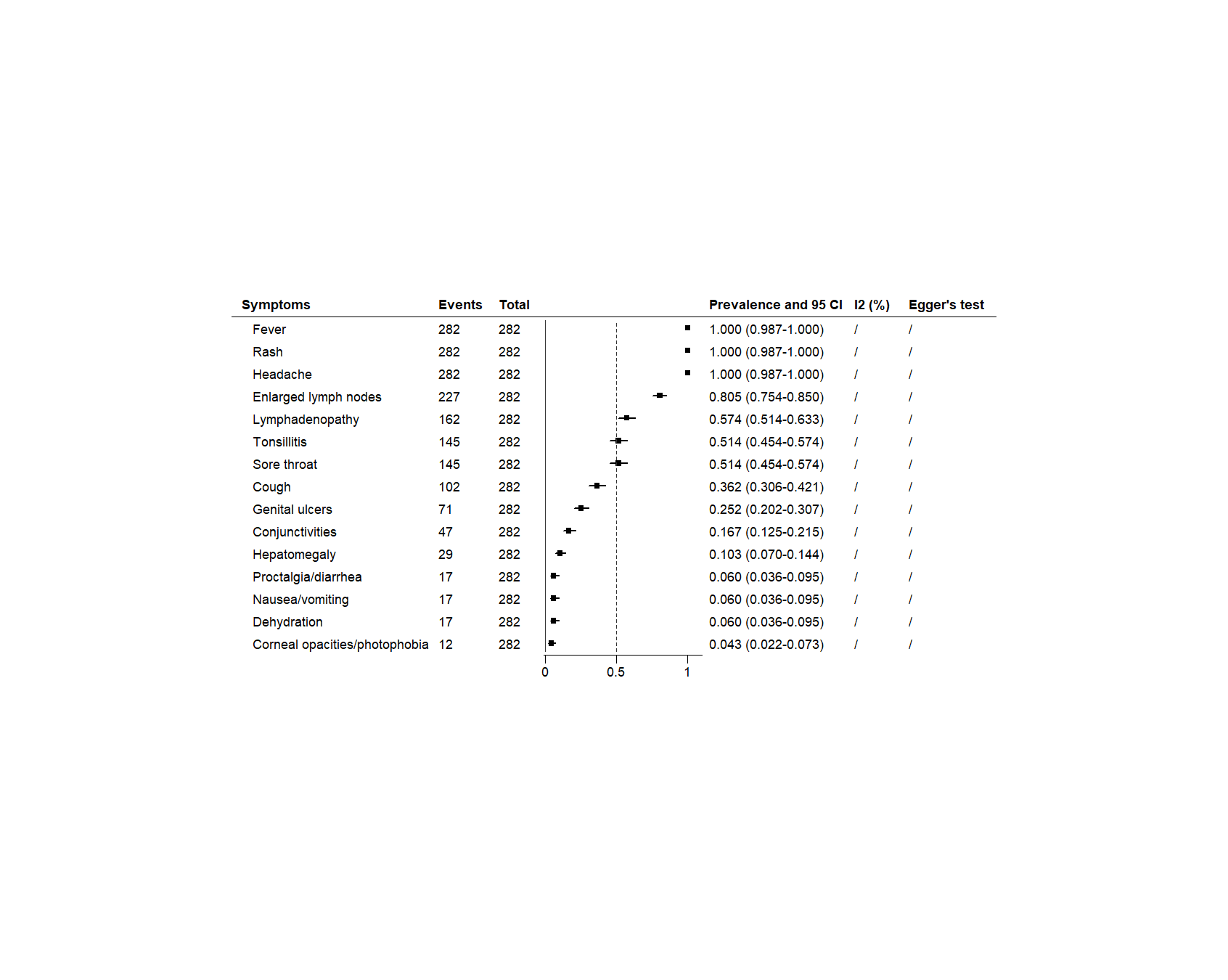


1. Sensitivity analysis from 1970-2002


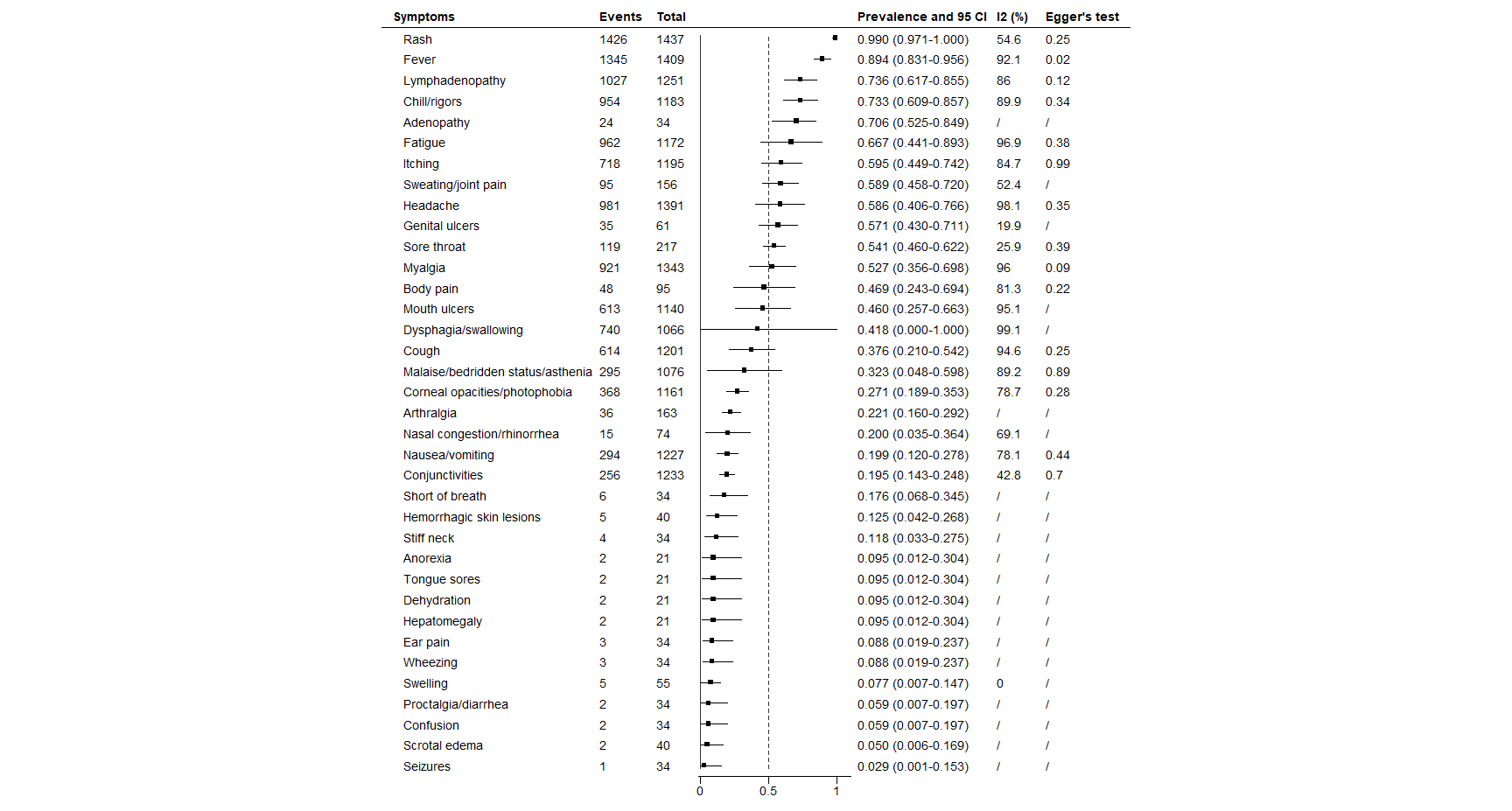


(b) Sensitivity analysis from 2003-2001


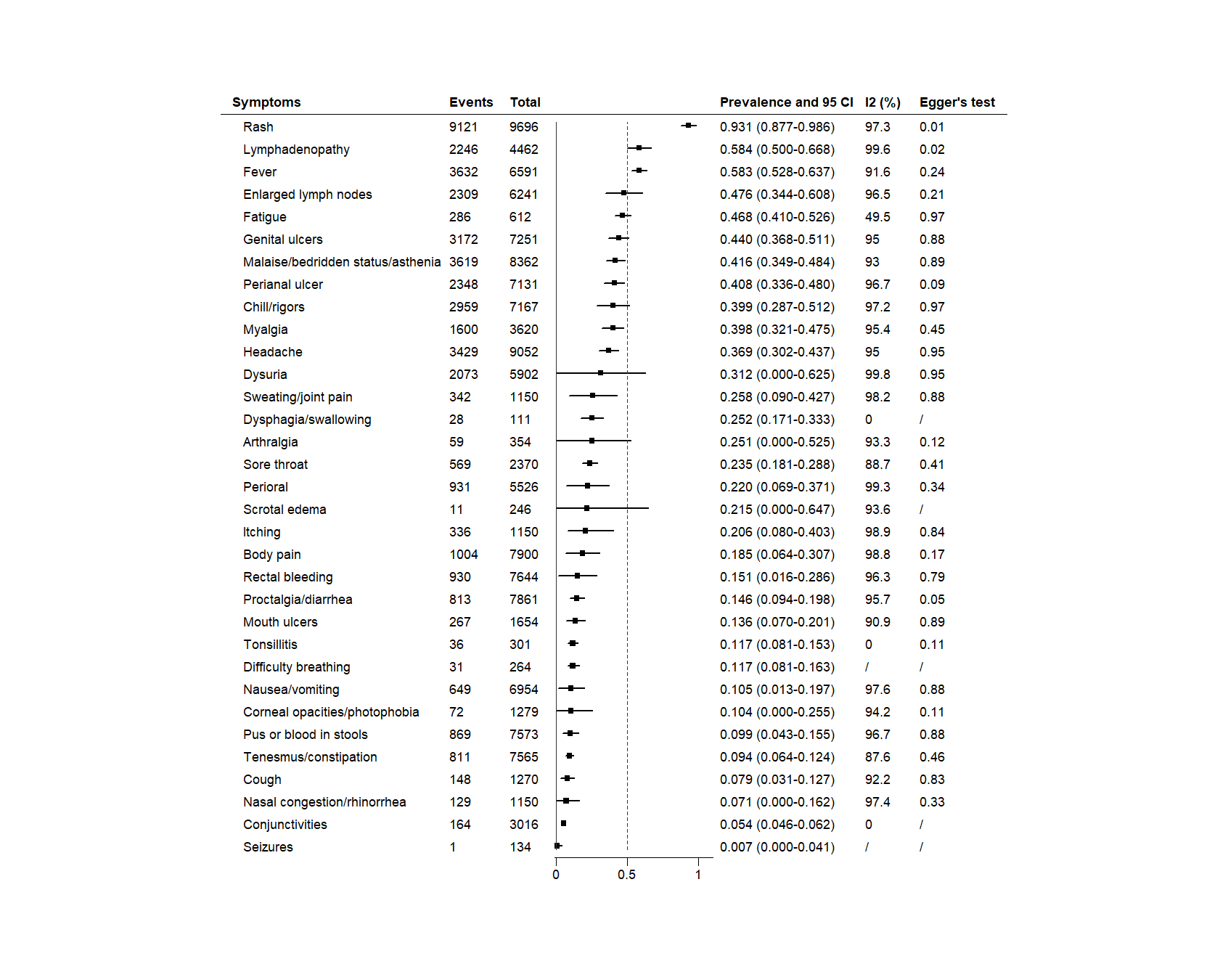


(c) Sensitivity analysis since 2022


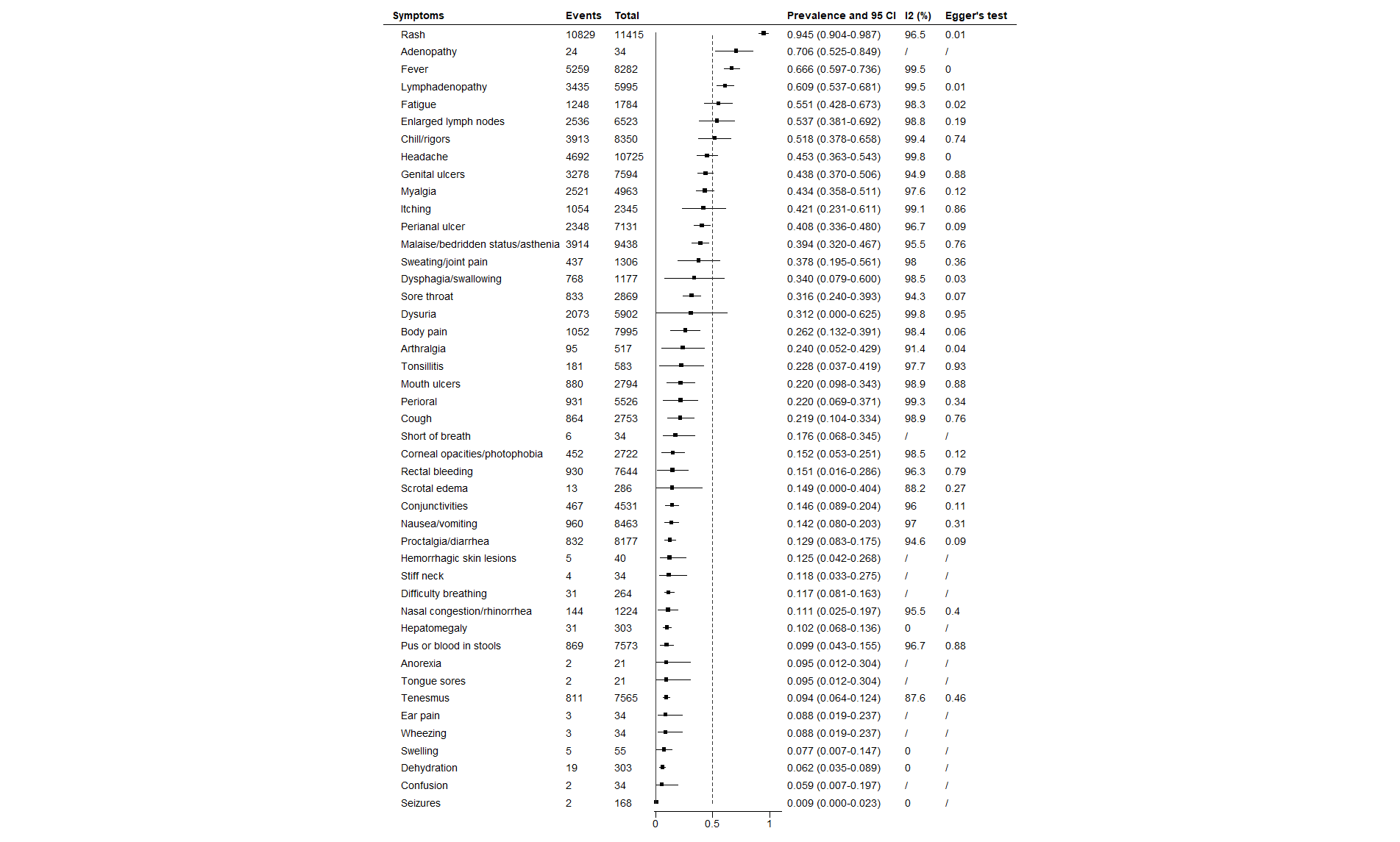


(d) Sensitivity analysis from 1970-2023

**Figure S3 Reported cases, death cases, and case fatality rate of monkeypox from 1970-2023**

1. Monkeypox cases from 1970-2023, a logarithmic scale
2. Case fatality rate of monkeypox cases from 1970-2023

1. Case fatality rate of monkeypox cases from main prevalence countries in 2022 and 2023

From 1970 to 2023, the number of reported cases has generally increased. The outbreak periods could be summarised in 1982-1986 (total 339 cases), 1995-1997 (543 cases), 2001-2013 (19872 cases), 2018-2021 (14370 cases), and 2022-2023 (84102 cases). High CFRs mainly occurred before 1986, 16.7% of CFR in 1970, 60.0% in 1972, 33.3% in 1973, 16.7% in 1975, 23.1% in 1978, and 9.7 – 12.5% from 1981-1985. From 1987-1995, no death cases were reported. After that, CFRs were lower than 6% between 1996 and 2022, the highest CFR emerged in 2016 (5.7%), followed by 2002 (4.2%), 2008 (4.2%) and 2001 (3.8%). In 2022, the CFR of MPX was decreased to 0.09%, while increased to 1.85 in 2023.The highest CFRs were occurred in Belgium (33.33%), Peru (7.64%), and United States (4.45%) in 2023 (Appendix Figure 3c). The MPX cases and CFR from 1970-2023 are described in Appendix Figure 3a and Appendix Figure 3b, respectively.

**Figure S4 Network diagram of the correlation between death and symptoms**


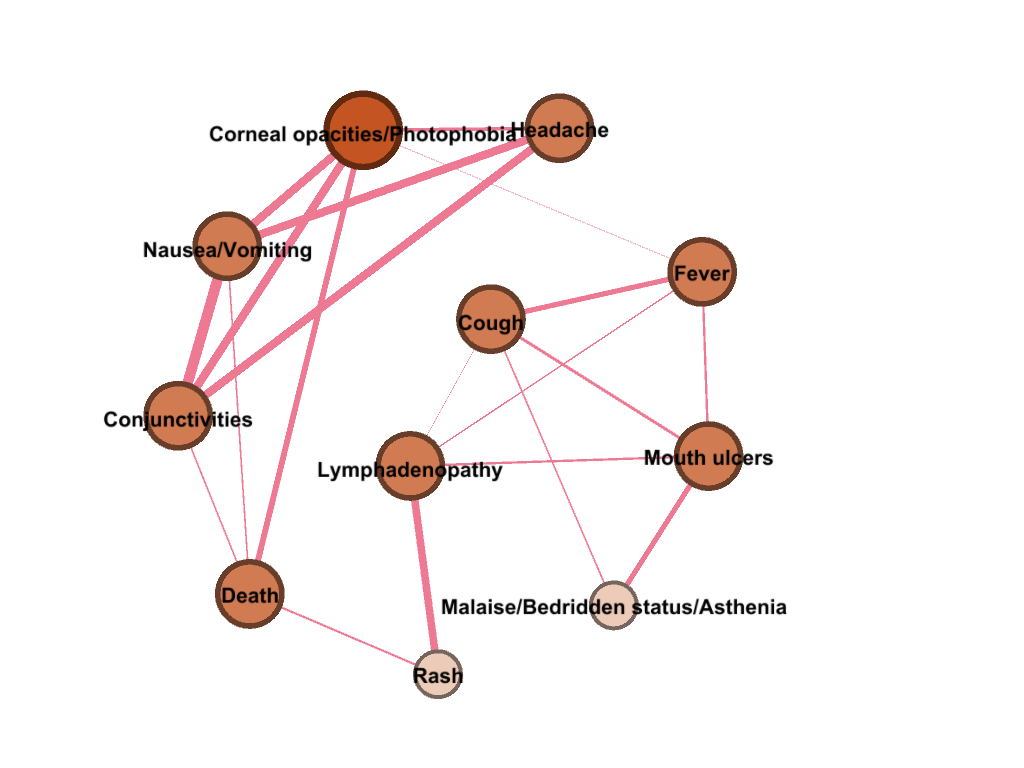


**Reference:**

1. Foster SO, Brink EW, Hutchins DL, Pifer JM, Lourie B, Moser CR, et al. Human monkeypox. Bull World Health Organ. 1972;46(5):569-76. PMID: 4340216.

2. Jezek Z, Szczeniowski M, Paluku KM, Mutombo M. Human monkeypox: clinical features of 282 patients. J Infect Dis. 1987 Aug;156(2):293-8. PMID: 3036967. doi: 10.1093/infdis/156.2.293.

3. Pebody R. Human Monkeypox in Kasai Oriental, Democratic Republic of Congo, February 1996 – October 1997: preliminary report. Weekly releases (1997–2007). 1997;1(32). doi: 10.2807/esw.01.32.01015-en.

4. Hutin Y, Williams RJ, Malfait P, Pebody R, Loparev VN, Ropp SL, et al. Outbreak of human monkeypox, Democratic Republic of Congo, 1996 to 1997. Emerging infectious diseases. 2001;7(3):434.

5. Huhn GD, Bauer AM, Yorita K, Graham MB, Sejvar J, Likos A, et al. Clinical characteristics of human monkeypox, and risk factors for severe disease. Clin Infect Dis. 2005 Dec 15;41(12):1742-51. PMID: 16288398. doi: 10.1086/498115.

6. Formenty P, Muntasir MO, Damon I, Chowdhary V, Opoka ML, Monimart C, et al. Human monkeypox outbreak caused by novel virus belonging to Congo Basin clade, Sudan, 2005. Emerg Infect Dis. 2010 Oct;16(10):1539-45. PMID: 20875278. doi: 10.3201/eid1610.100713.

7. Whitehouse ER, Bonwitt J, Hughes CM, Lushima RS, Likafi T, Nguete B, et al. Clinical and Epidemiological Findings from Enhanced Monkeypox Surveillance in Tshuapa Province, Democratic Republic of the Congo During 2011-2015. J Infect Dis. 2021 Jun 4;223(11):1870-8. PMID: 33728469. doi: 10.1093/infdis/jiab133.

8. Singapore Zika Study G. Outbreak of Zika virus infection in Singapore: an epidemiological, entomological, virological, and clinical analysis. Lancet Infect Dis. 2017 Aug;17(8):813-21. PMID: 28527892. doi: 10.1016/S1473-3099(17)30249-9.

9. Yinka-Ogunleye A, Aruna O, Dalhat M, Ogoina D, McCollum A, Disu Y, et al. Outbreak of human monkeypox in Nigeria in 2017-18: a clinical and epidemiological report. Lancet Infect Dis. 2019 Aug;19(8):872-9. PMID: 31285143. doi: 10.1016/S1473-3099(19)30294-4.

10. Ogoina D, Izibewule JH, Ogunleye A, Ederiane E, Anebonam U, Neni A, et al. The 2017 human monkeypox outbreak in Nigeria-Report of outbreak experience and response in the Niger Delta University Teaching Hospital, Bayelsa State, Nigeria. PLoS One. 2019;14(4):e0214229. PMID: 30995249. doi: 10.1371/journal.pone.0214229.

11. Ogoina D, Iroezindu M, James HI, Oladokun R, Yinka-Ogunleye A, Wakama P, et al. Clinical Course and Outcome of Human Monkeypox in Nigeria. Clin Infect Dis. 2020 Nov 5;71(8):e210-e4. PMID: 32052029. doi: 10.1093/cid/ciaa143.

12. Vaughan A, Aarons E, Astbury J, Balasegaram S, Beadsworth M, Beck CR, et al. Two cases of monkeypox imported to the United Kingdom, September 2018. Euro Surveill. 2018 Sep;23(38). PMID: 30255836. doi: 10.2807/1560-7917.ES.2018.23.38.1800509.

13. Yong SEF, Ng OT, Ho ZJM, Mak TM, Marimuthu K, Vasoo S, et al. Imported Monkeypox, Singapore. Emerg Infect Dis. 2020 Aug;26(8):1826-30. PMID: 32338590. doi: 10.3201/eid2608.191387.

14. Tumewu J, Wardiana M, Ervianty E, Sawitri, Rahmadewi, Astindari, et al. An adult patient with suspected of monkeypox infection differential diagnosed to chickenpox. Infect Dis Rep. 2020 Jul 7;12(Suppl 1):8724. PMID: 32874456. doi: 10.4081/idr.2020.8724.

15. Hobson G, Adamson J, Adler H, Firth R, Gould S, Houlihan C, et al. Family cluster of three cases of monkeypox imported from Nigeria to the United Kingdom, May 2021. Euro Surveill. 2021 Aug;26(32). PMID: 34387184. doi: 10.2807/1560-7917.ES.2021.26.32.2100745.

16. Rao AK, Schulte J, Chen TH, Hughes CM, Davidson W, Neff JM, et al. Monkeypox in a Traveler Returning from Nigeria - Dallas, Texas, July 2021. MMWR Morb Mortal Wkly Rep. 2022 Apr 8;71(14):509-16. PMID: 35389974. doi: 10.15585/mmwr.mm7114a1.

17. Catala A, Clavo-Escribano P, Riera-Monroig J, Martin-Ezquerra G, Fernandez-Gonzalez P, Revelles-Penas L, et al. Monkeypox outbreak in Spain: clinical and epidemiological findings in a prospective cross-sectional study of 185 cases. Br J Dermatol. 2022 Nov;187(5):765-72. PMID: 35917191. doi: 10.1111/bjd.21790.

18. Cassir N, Cardona F, Tissot-Dupont H, Bruel C, Doudier B, Lahouel S, et al. Observational Cohort Study of Evolving Epidemiologic, Clinical, and Virologic Features of Monkeypox in Southern France. Emerg Infect Dis. 2022 Dec;28(12):2409-15. PMID: 36241422. doi: 10.3201/eid2812.221440.

19. de Sousa D, Patrocínio J, Frade J, Brazão C, Mancha D, Correia C, et al. Monkeypox Diagnosis by Cutaneous and Mucosal Findings. Infectious Disease Reports. 2022;14(5):759-64. doi: 10.3390/idr14050077.

20. Girometti N, Byrne R, Bracchi M, Heskin J, McOwan A, Tittle V, et al. Demographic and clinical characteristics of confirmed human monkeypox virus cases in individuals attending a sexual health centre in London, UK: an observational analysis. Lancet Infect Dis. 2022 Sep;22(9):1321-8. PMID: 35785793. doi: 10.1016/S1473-3099(22)00411-X.

21. Martins-Filho PR, de Souza MF, Oliveira Gois MA, Bezerra GVB, Goncalves CCA, Dos Santos Nascimento ER, et al. Unusual epidemiological presentation of the first reports of monkeypox in a low-income region of Brazil. Travel Med Infect Dis. 2022 Nov-Dec;50:102466. PMID: 36180021. doi: 10.1016/j.tmaid.2022.102466.

22. Patel A, Bilinska J, Tam JCH, Da Silva Fontoura D, Mason CY, Daunt A, et al. Clinical features and novel presentations of human monkeypox in a central London centre during the 2022 outbreak: descriptive case series. BMJ. 2022 Jul 28;378:e072410. PMID: 35902115. doi: 10.1136/bmj-2022-072410.

23. Philpott D, Hughes CM, Alroy KA, Kerins JL, Pavlick J, Asbel L, et al. Epidemiologic and Clinical Characteristics of Monkeypox Cases - United States, May 17-July 22, 2022. MMWR Morb Mortal Wkly Rep. 2022 Aug 12;71(32):1018-22. PMID: 35951487. doi: 10.15585/mmwr.mm7132e3.

24. Thornhill JP, Barkati S, Walmsley S, Rockstroh J, Antinori A, Harrison LB, et al. Monkeypox Virus Infection in Humans across 16 Countries - April-June 2022. N Engl J Med. 2022 Aug 25;387(8):679-91. PMID: 35866746. doi: 10.1056/NEJMoa2207323.

25. Tarin-Vicente EJ, Alemany A, Agud-Dios M, Ubals M, Suner C, Anton A, et al. Clinical presentation and virological assessment of confirmed human monkeypox virus cases in Spain: a prospective observational cohort study. Lancet. 2022 Aug 27;400(10353):661-9. PMID: 35952705. doi: 10.1016/S0140-6736(22)01436-2.

26. Suner C, Ubals M, Tarin-Vicente EJ, Mendoza A, Alemany A, Hernandez-Rodriguez A, et al. Viral dynamics in patients with monkeypox infection: a prospective cohort study in Spain. Lancet Infect Dis. 2023 Apr;23(4):445-53. PMID: 36521505. doi: 10.1016/S1473-3099(22)00794-0.

27. Caria J, Pinto R, Leal E, Almeida V, Cristovao G, Goncalves AC, et al. Clinical and Epidemiological Features of Hospitalized and Ambulatory Patients with Human Monkeypox Infection: A Retrospective Observational Study in Portugal. Infect Dis Rep. 2022 Oct 27;14(6):810-23. PMID: 36412741. doi: 10.3390/idr14060083.

28. Roy SF, Sarhan J, Liu X, Murphy MJ, Bunick CG, Choate KA, et al. Inguinal patch in mpox (monkeypox) virus infection and eccrine syringometaplasia: report of two cases with in situ hybridization and electron microscopy findings. Br J Dermatol. 2023 Mar 30;188(4):574-6. PMID: 36763786. doi: 10.1093/bjd/ljac146.

29. Sihuincha Maldonado M, Lucchetti AJ, Paredes Pacheco RA, Martinez Cevallos LC, Zumaeta Saavedra EU, Ponce Zapata LR, et al. Epidemiologic characteristics and clinical features of patients with monkeypox virus infection from a hospital in Peru between July and September 2022. Int J Infect Dis. 2023 Apr;129:175-80. PMID: 36740013. doi: 10.1016/j.ijid.2023.01.045.

30. Prasad S, Galvan Casas C, Strahan AG, Fuller LC, Peebles K, Carugno A, et al. A dermatologic assessment of 101 mpox (monkeypox) cases from 13 countries during the 2022 outbreak: Skin lesion morphology, clinical course, and scarring. J Am Acad Dermatol. 2023 May;88(5):1066-73. PMID: 36641010. doi: 10.1016/j.jaad.2022.12.035.

31. Martinez CAP, Flores GAS, Santamaria FP, Franco LM, Cano FF, Fierro LAG, et al. Monkeypox and its broad clinical spectrum in immunocompromised patients: Two case reports. IDCases. 2023;31:e01651. PMID: 36465316. doi: 10.1016/j.idcr.2022.e01651.

32. Patalon T, Perez G, Melamed G, Wolf T, Gazit S. Mpox Infection in a Developed Country: A Case Report. Trop Med Infect Dis. 2022 Dec 27;8(1). PMID: 36668922. doi: 10.3390/tropicalmed8010015.

33. Proietti I, Santoro PE, Skroza N, Tieghi T, Bernardini N, Tolino E, et al. A Case Report of Monkeypox in an Adult Patient from Italy: Clinical and Dermoscopic Manifestations, Diagnosis and Management. Vaccines (Basel). 2022 Nov 10;10(11). PMID: 36366410. doi: 10.3390/vaccines10111903.

34. Manoharan A, Braz BX, McBride A, Hernandez S, Balfour M, Quiroz T, et al. Severe monkeypox with superimposed bacterial infection in an immunocompetent patient: A case report. IDCases. 2022;30:e01626. PMID: 36345426. doi: 10.1016/j.idcr.2022.e01626.

35. Pisano L, Turco M, Mancuso FR, Lastrucci I, Pimpinelli N. Atypical oral presentation of monkeypox virus: A report of two cases from Florence, Italy. Travel Med Infect Dis. 2022 Nov-Dec;50:102457. PMID: 36113755. doi: 10.1016/j.tmaid.2022.102457.

36. Cutoiu A, Boda D. Monkeypox 2022: Dermatologists in the frontline on the edge of a new pandemic: A case report. Exp Ther Med. 2022 Nov;24(5):677. PMID: 36185764. doi: 10.3892/etm.2022.11613.

37. Desgranges F, Glampedakis E, Christinet V, Encarnacao S, Fernandes C, Greub G, et al. Rectal shedding of monkeypox virus in a patient coinfected with Chlamydia trachomatis and Neisseria gonorrhoeae: a case report. J Med Case Rep. 2023 Mar 6;17(1):94. PMID: 36872313. doi: 10.1186/s13256-023-03826-z.

38. Angelo KM, Smith T, Camprubi-Ferrer D, Balerdi-Sarasola L, Diaz Menendez M, Servera-Negre G, et al. Epidemiological and clinical characteristics of patients with monkeypox in the GeoSentinel Network: a cross-sectional study. Lancet Infect Dis. 2023 Feb;23(2):196-206. PMID: 36216018. doi: 10.1016/S1473-3099(22)00651-X.

39. Zayat N, Huang S, Wafai J, Philadelphia M. Monkeypox Virus Infection in 22-Year-Old Woman after Sexual Intercourse, New York, USA. Emerg Infect Dis. 2023 Jan;29(1):222-3. PMID: 36355634. doi: 10.3201/eid2901.221662.

40. Berens-Riha N, De Block T, Rutgers J, Michiels J, Van Gestel L, Hens M, et al. Severe mpox (formerly monkeypox) disease in five patients after recent vaccination with MVA-BN vaccine, Belgium, July to October 2022. Euro Surveill. 2022 Dec;27(48). PMID: 36695462. doi: 10.2807/1560-7917.ES.2022.27.48.2200894.

41. Oakley LP, Hufstetler K, O'Shea J, Sharpe JD, McArdle C, Neelam V, et al. Mpox Cases Among Cisgender Women and Pregnant Persons - United States, May 11-November 7, 2022. MMWR Morb Mortal Wkly Rep. 2023 Jan 6;72(1):9-14. PMID: 36602932. doi: 10.15585/mmwr.mm7201a2.

42. Farrar JL, Lewis NM, Houck K, Canning M, Fothergill A, Payne AB, et al. Demographic and Clinical Characteristics of Mpox in Persons Who Had Previously Received 1 Dose of JYNNEOS Vaccine and in Unvaccinated Persons - 29 U.S. Jurisdictions, May 22-September 3, 2022. MMWR Morb Mortal Wkly Rep. 2022 Dec 30;71(5152):1610-5. PMID: 36580416. doi: 10.15585/mmwr.mm715152a2.

43. Kyaw NTT, Kipperman N, Alroy KA, Baumgartner J, Crawley A, Peterson E, et al. Notes from the Field: Clinical and Epidemiologic Characteristics of Mpox Cases from the Initial Phase of the Outbreak - New York City, May 19-July 15, 2022. MMWR Morb Mortal Wkly Rep. 2022 Dec 30;71(5152):1631-3. PMID: 36580429. doi: 10.15585/mmwr.mm715152a3.

44. Assiri AM, Al-Tawfiq JA, Jokhdar HA, Algwizani AR, Albarraq AM, Alanazi KH, et al. Clinical features and outcome of human Mpox (Monkeypox) in Saudi Arabia: An observational study of travel-related cases. J Infect Public Health. 2023 Mar;16(3):341-5. PMID: 36680849. doi: 10.1016/j.jiph.2023.01.006.

45. Palich R, Burrel S, Monsel G, Nouchi A, Bleibtreu A, Seang S, et al. Viral loads in clinical samples of men with monkeypox virus infection: a French case series. Lancet Infect Dis. 2023 Jan;23(1):74-80. PMID: 36183707. doi: 10.1016/S1473-3099(22)00586-2.

46. Alpalhao M, Sousa D, Frade JV, Patrocinio J, Garrido PM, Correia C, et al. Human immunodeficiency virus infection may be a contributing factor to monkeypox infection: Analysis of a 42-case series. J Am Acad Dermatol. 2023 Mar;88(3):720-2. PMID: 36156305. doi: 10.1016/j.jaad.2022.09.029.

47. Rimmer S, Barnacle J, Gibani MM, Wu MS, Dissanayake O, Mehta R, et al. The clinical presentation of monkeypox: a retrospective case-control study of patients with possible or probable monkeypox in a West London cohort. Int J Infect Dis. 2023 Jan;126:48-53. PMID: 36410691. doi: 10.1016/j.ijid.2022.11.020.

48. Jang W, Kandimalla L, Rajan S, Abreu R, Campos JE. Monkeypox in an immunocompromised patient with underlying human immunodeficiency virus and syphilis infections in Southern Florida of the United States: a case report. AIDS Res Ther. 2023 Feb 18;20(1):12. PMID: 36800970. doi: 10.1186/s12981-023-00504-4.

49. Huang ST, Wu YH, Lin HH, Yang JY, Hsieh PY, Chiang SJ, et al. The first imported case of monkeypox in Taiwan. J Formos Med Assoc. 2023 Jan;122(1):73-7. PMID: 36175217. doi: 10.1016/j.jfma.2022.08.014.

50. Vallee A, Chatelain A, Carbonnel M, Racowsky C, Fourn E, Zucman D, et al. Monkeypox Virus Infection in 18-Year-Old Woman after Sexual Intercourse, France, September 2022. Emerg Infect Dis. 2023 Jan;29(1):219-22. PMID: 36355630. doi: 10.3201/eid2901.221643.

51. Hennessee I, Shelus V, McArdle CE, Wolf M, Schatzman S, Carpenter A, et al. Epidemiologic and Clinical Features of Children and Adolescents Aged <18 Years with Monkeypox - United States, May 17-September 24, 2022. MMWR Morb Mortal Wkly Rep. 2022 Nov 4;71(44):1407-11. PMID: 36331124. doi: 10.15585/mmwr.mm7144a4.

52. Thornhill JP, Palich R, Ghosn J, Walmsley S, Moschese D, Cortes CP, et al. Human monkeypox virus infection in women and non-binary individuals during the 2022 outbreaks: a global case series. Lancet. 2022 Dec 3;400(10367):1953-65. PMID: 36403584. doi: 10.1016/S0140-6736(22)02187-0.

53. Choudhury K, Eberhardt KA, Haugk R, Holtz N, Claass J, Duwe J, et al. The First 179 Cases of Monkeypox in Hamburg-Demographic, Temporal, and Geographic Distribution. Dtsch Arztebl Int. 2022 Nov 11;119(45):773-4. PMID: 36700369. doi: 10.3238/arztebl.m2022.0340.

54. Pipito L, Cascio A. Monkeypox virus infection and creatine phosphokinase increase: A case from Italy. Travel Med Infect Dis. 2022 Nov-Dec;50:102412. PMID: 35970435. doi: 10.1016/j.tmaid.2022.102412.

55. Lopes PS, Haddad GR, Miot HA. Sexually-transmitted monkeypox: report of two cases. Anais Brasileiros de Dermatologia. 2022;97(6):783-5. doi: 10.1016/j.abd.2022.08.002.

56. Mailhe M, Beaumont AL, Thy M, Le Pluart D, Perrineau S, Houhou-Fidouh N, et al. Clinical characteristics of ambulatory and hospitalized patients with monkeypox virus infection: an observational cohort study. Clin Microbiol Infect. 2023 Feb;29(2):233-9. PMID: 36028090. doi: 10.1016/j.cmi.2022.08.012.

57. Noe S, Zange S, Seilmaier M, Antwerpen MH, Fenzl T, Schneider J, et al. Clinical and virological features of first human monkeypox cases in Germany. Infection. 2023 Feb;51(1):265-70. PMID: 35816222. doi: 10.1007/s15010-022-01874-z.

58. Nouchi A, Brin C, Martin A, Favier M, Palich R, Wakim Y, et al. Prospective cohort of 70 consecutive cases of human monkeypox: Clinical description with focus on dermatological presentation. J Eur Acad Dermatol Venereol. 2023 Mar;37(3):e403-e5. PMID: 36377334. doi: 10.1111/jdv.18742.

59. Srichawla BS, Garcia-Dominguez MA, Zia S. Secondary complications and management strategies in human monkeypox: A case series. J Med Virol. 2023 Feb;95(2):e28449. PMID: 36583470. doi: 10.1002/jmv.28449.

60. Garcia-Piqueras P, Bergon-Sendin M, Cordoba-Garcia-Rayo M, Virseda-Gonzalez D, Medrano-Martinez N, Jimenez-Briones L, et al. Human monkeypox virus in a tertiary hospital in Madrid, Spain: An observational study of the clinical and epidemiological characteristics of 53 cases. Exp Dermatol. 2023 Feb;32(2):198-202. PMID: 36222009. doi: 10.1111/exd.14687.

61. Rekik S, Pluart DL, Ferré V, Charpentier C, Laurain A, Ghosn J, et al. Anogenital symptoms and lesions in a series of 20 patients infected with monkeypox virus. Colorectal Disease. 2023. doi: 10.1111/codi.16513.

62. J. G. BREMAN, KALISA-RUTI, M. V. STENIOWSKI, E. ZANOTTO, A. I. GROMYKO, ARITA I. Monkeypox chez 1'homme, 1970-79. Bulletin de l'Organisation mondiale de la Sante,. 1980;58(6):849-68.

63. Z. JEZEK, L. N. KHODAKEVICH, WICKETTI JF. Smallpox and its post-eradication surveillance. Buletin of the World Health Organization. 1987;65(4):425-34.

64. The current status of human monkeypox: Memorandum from a WHO Meeting. Bulletin ofthe World Health Organization. 1984;62(5):703-13.

65. F JZ. Human monkeypox. Monographs in Virology. Karger, editor. Basel1988.

66. Di Giulio DB, Eckburg PB. Human monkeypox: an emerging zoonosis. The Lancet Infectious Diseases. 2004;4(1):15-25. doi: 10.1016/s1473-3099(03)00856-9.

67. Beer EM, Rao VB. A systematic review of the epidemiology of human monkeypox outbreaks and implications for outbreak strategy. PLoS Negl Trop Dis. 2019 Oct;13(10):e0007791. PMID: 31618206. doi: 10.1371/journal.pntd.0007791.

68. Yvan J.F. Hutin, R. Joel Williams, Philippe Malfait, Richard Pebody, Vladamir N. Loparev, Susan L. Ropp, et al. Outbreak of Human Monkeypox, Democratic Republic of Congo, 1996–1997. Emerging Infectious Diseases. 2001;7(3):434-8.

69. Fine PEM, Jezek Z, Grab B, Dixon H. The Transmission Potential of Monkeypox Virus in Human Populations. International journal of epidemiology. 1988;17(3):643-50. doi: 10.1093/ije/17.3.643.

70. Hoff N, Ilunga BK, Shongo R, Muyembe JJ, Mossoko M, Okitolonda E ea. Human monkeypox disease surveillance and time trends in The Democratic Republic of Congo, 2001-2013. American Journal of Tropical Medicine and Hygiene. 2014;(1):339.

71. Berthet N, Nakouné E, Whist E, Selekon B, Burguière AM, Manuguerra JC, et al. Maculopapular lesions in the Central African Republic. Lancet. 2011;378(9799):1354.

72. Learned LA, Reynolds MG, Wassa DW, Li Y, Olson VA, Karem K, et al. Extended interhuman transmission of monkeypox in a hospital community in the Republic of the Congo, 2003. . Am J Trop Med Hyg. 2005;73(2):428-34.

73. IRIN. Monkeypox infects 60 in north. Available online at: <http://wwwirinnewsorg/report/74469/congo-monkeypox-infects-60-north> (Accessed 17/01/2017). 2007.

74. Reynolds MG, Emerson GL, Pukuta E, Karhemere S, Muyembe JJ, Bikindou A, et al. Detection of human monkeypox in the Republic of the Congo following intensive community education. Am J Trop Med Hyg. 2013 May;88(5):982-5. PMID: 23400570. doi: 10.4269/ajtmh.12-0758.

75. RadioOkapi. Bas-Uélé : 51 nouveaux cas de Monkey Pox enregistrés, 2 morts. Available online at: <http://wwwradiookapinet/2016/02/10/actualite/sante/bas-uele-51-nouveaux-cas-de-monkey-pox-enregistres-2-morts> (Accessed 20/01/2017). 2016.

76. Sklenovska N, Van Ranst M. Emergence of Monkeypox as the Most Important Orthopoxvirus Infection in Humans. Front Public Health. 2018;6:241. PMID: 30234087. doi: 10.3389/fpubh.2018.00241.

77. WHO. Weekly Bulletin on Outbreaks and Other Emergencies, week 8 2018. Available online at: <http://appswhoint/iris/bitstream/10665/260335/1/OEW8-1723022018pdf> (Accessed 22/03/2018). 2018.

78. WHO. Weekly Bulletin on Outbreaks and Other Emergencies, week 48 2017. Available online at: <http://appswhoint/iris/bitstream/10665/259557/1/OEW48-2504122017pdf> (accessed 22/03/2018). 2017.

79. Amao LK, Olatunji DI, Igbodo G, Okoli SC, Amaechi I, Goni MI, et al. Trend and enhanced surveillance of Monkeypox during COVID-19 pandemic in Nigeria. J Public Health Afr. 2022 May 24;13(1):2184. PMID: 35720796. doi: 10.4081/jphia.2022.2184.

80. WHO. Monkeypox - Democratic Republic of the Congo. <https://wwwwhoint/emergencies/disease-outbreak-news/item/monkeypox-democratic-republic-of-the-congo>. 2020.

81. Erez N, Achdout H, Milrot E, Schwartz Y, Wiener-Well Y, Paran N, et al. Diagnosis of Imported Monkeypox, Israel, 2018. Emerg Infect Dis. 2019 May;25(5):980-3. PMID: 30848724. doi: 10.3201/eid2505.190076.

82. Ng OT, Lee V, Marimuthu K, Vasoo S, Chan G, Lin RTP, et al. A case of imported Monkeypox in Singapore. The Lancet Infectious Diseases. 2019;19(11). doi: 10.1016/s1473-3099(19)30537-7.

83. WHO. Provisional monthly measles and rubella data. [2021–12–17] <https://wwwwhoint/teams/immunization-vaccines-and-biologicals/immunizationanalysis-and-insights/surveillance/monitoring/provisional-monthlymeasles-and-rubella-data>. 2021.

84. Mathieu E, Spooner F, Dattani S, Ritchie H, Roser M. "Monkeypox". Published online at OurWorldInData.org. Retrieved from: '<https://ourworldindata.org/monkeypox'> [Online Resource]2022.

85. Bank W. Population, total. 2022.

86. STANLEY O. FOSTER, EDWARD W. BRINK, DEANE L. HUTCHINS, JOHN M. PIFER, BERNARD LOURIE, CLAUDE R. MOSER ECC, et al. Human monkeypox. Bull Org mond Santie & Bull Wld Hith Org. 1972;46.
